# ARID1A-BAF coordinates ZIC2 genomic occupancy for epithelial-to-mesenchymal transition in cranial neural crest specification

**DOI:** 10.1016/j.ajhg.2024.07.022

**Published:** 2024-09-02

**Authors:** Samantha M. Barnada, Aida Giner de Gracia, Cruz Morenilla-Palao, Maria Teresa López-Cascales, Chiara Scopa, Francis J. Waltrich, Harald M.M. Mikkers, Maria Elena Cicardi, Jonathan Karlin, Davide Trotti, Kevin A. Peterson, Samantha A. Brugmann, Gijs W.E. Santen, Steven B. McMahon, Eloísa Herrera, Marco Trizzino

**Affiliations:** 1 Department of Biochemistry and Molecular Biology, Thomas Jefferson University, Philadelphia, PA, USA; 2 Department of Life Sciences, Imperial College London, London, UK; 3 Instituto de Neurociencias de Alicante (Consejo Superior de Investigaciones Científicas- Universidad Miguel Hernández, CSIC-UMH). Campus San Juan, Avd. Ramón y Cajal s/n, 03550 San Juan de Alicante, Spain; 4 Jefferson Weinberg ALS Center, Vickie and Jack Farber Institute for Neuroscience, Department of Neuroscience, Thomas Jefferson University, Philadelphia, PA, USA; 5 Department of Pharmacology, Physiology, and Cancer Biology, Thomas Jefferson University, Philadelphia, PA 19107, USA; 6 Department of Cell & Chemical Biology, Leiden University Medical Center, Leiden, the Netherlands; 7 The Jackson Laboratory, 600 Main St, Bar Harbor, ME 04609, USA; 8 Division of Developmental Biology, Department of Pediatrics at Cincinnati Children’s Hospital Medical Center, Cincinnati, OH, USA; 9 Department of Clinical Genetics, Leiden University Medical Center, Leiden, the Netherlands

**Keywords:** ARID1A, BAF, SWI/SNF, cranial neural crest, iPSCs, Coffin-Siris syndrome, holoprosencephaly type 5, ZIC2, epithelial-to-mesenchymal transition

## Abstract

The BAF chromatin remodeler regulates lineage commitment including cranial neural crest cell (CNCC) specification. Variants in BAF subunits cause Coffin-Siris syndrome (CSS), a congenital disorder characterized by coarse craniofacial features and intellectual disability. Approximately 50% of individuals with CSS harbor variants in one of the mutually exclusive BAF subunits, *ARID1A/ARID1B*. While *Arid1a* deletion in mouse neural crest causes severe craniofacial phenotypes, little is known about the role of ARID1A in CNCC specification. Using CSS-patient-derived *ARID1A*^+/−^ induced pluripotent stem cells to model CNCC specification, we discovered that *ARID1A*-haploinsufficiency impairs epithelial-to-mesenchymal transition (EMT), a process necessary for CNCC delamination and migration from the neural tube. Furthermore, wild-type ARID1A-BAF regulates enhancers associated with EMT genes. ARID1A-BAF binding at these enhancers is impaired in heterozygotes while binding at promoters is unaffected. At the sequence level, these EMT enhancers contain binding motifs for ZIC2, and ZIC2 binding at these sites is ARID1A-dependent. When excluded from EMT enhancers, ZIC2 relocates to neuronal enhancers, triggering aberrant neuronal gene activation. In mice, deletion of *Zic2* impairs NCC delamination, while *ZIC2* overexpression in chick embryos at post-migratory neural crest stages elicits ectopic delamination from the neural tube. These findings reveal an essential ARID1A-ZIC2 axis essential for EMT and CNCC delamination.

## Graphical abstract

**Figure:**
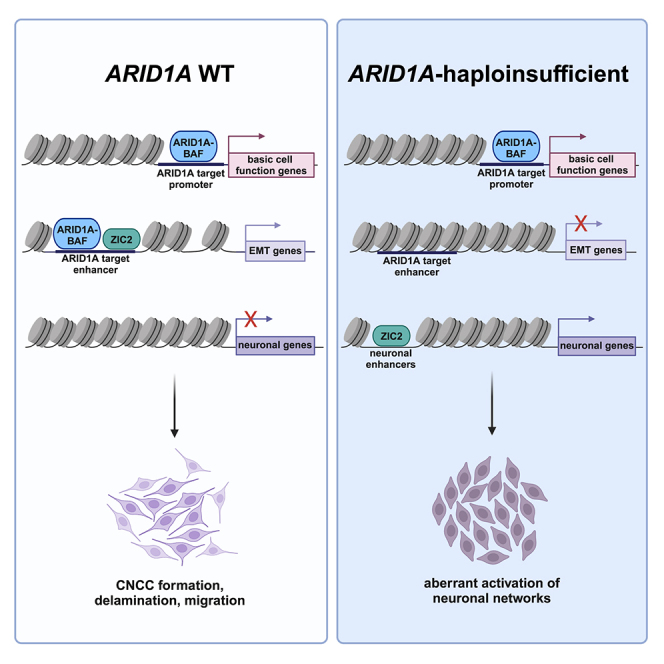

## Teaser

ARID1A modulates chromatin accessibility at enhancers of genes required for epithelial-to-mesenchymal transition, a process essential for cranial neural crest cell (CNCC) specification. Haploinsufficiency of *ARID1A* attenuates ZIC2 binding at these enhancers, resulting in impaired CNCC formation with an aberrant neuronal trajectory. This study reveals an ARID1A-ZIC2 axis essential for CNCC specification.

## Introduction

The mammalian SWI/SNF complex, referred to as BRG1/BRM associated factor (BAF), is an ATP-dependent chromatin remodeler.^1^ BAF plays global roles in lineage specification,^2^^,^^3^^,^^4^ pluripotency,^5^ tumorigenesis,^6^ and basic cellular processes^7^^,^^8^^,^^9^ by modulating chromatin accessibility and interacting with transcription factors^10^ to impact gene regulation. BAF comprises three main modules: (1) the ATPase module that hydrolyzes ATP for catalytic activity to alter nucleosome positioning, (2) the ARP module, which aids in ATPase module functions, and (3) the core module required for complex assembly, stabilization, and DNA binding.^11^^,^^12^ The three BAF subtypes, canonical BAF, polybromo-associated BAF (PBAF), and non-canonical (GLTSCR1/1L-containing) BAF (GBAF)^5^^,^^13^ include all the main modules in varying configurations that contain interchangeable subunits that confer context-dependent functions.^14^^,^^15^^,^^16^ Canonical BAF (hereafter “BAF”) is the only subtype that contains the large, mutually exclusive AT-rich interaction domain 1A and 1B (ARID1A/ARID1B) subunits in its core module.^11^^,^^12^^,^^15^

ARID1A and ARID1B are necessary for BAF complex formation, stability, and DNA binding.^12^^,^^15^^,^^16^ *De novo* alterations in *ARID1A* can occur at all stages from germline mutations to somatic mosaicism in embryonic development^17^^,^^18^ and adult tissue.^6^^,^^19^ In cancer, ARID1A is reported to have context-dependent oncogenic^20^^,^^21^ and/or tumor suppressive functions^22^^,^^23^^,^^24^^,^^25^ as variants in *ARID1A* occur in ∼10% of all tumors^6^^,^^26^ and ∼50% of specific cancer types.^24^^,^^27^ In a developmental setting, heterozygous loss-of-function variants in *ARID1A* (as well as variants in other BAF subunits) are causative of Coffin-Siris syndrome (CSS)^28^^,^^29^ due to haploinsufficiency. CSS is a rare developmental disorder characterized by systemic congenital anomalies, including distal limb phenotypes, intellectual disability, and coarse craniofacial features.^30^^,^^31^^,^^32^ These dysmorphic craniofacial features, including a depressed nasal bridge, short nose, averted nares, broad philtrum, and wide mouth,^32^ result from impaired craniofacial development.

The process of forming the craniofacial skeleton relies on complex spatiotemporal regulation and patterning and includes derivatives of all three germ layers and neural crest cells (NCCs).^33^ NCCs are a transient cell population that arise between weeks 3 and 4 of human embryonic development during neurulation.^34^ Post-gastrulation, the dorsal portion of the ectoderm begins to specify into neuroectoderm, forming the neural plate that is flanked by the neural plate border.^34^ During neurulation, the neural plate invaginates and separates from the dorsal non-neural ectoderm at the neural plate border to form the neural tube.^34^ At this neural plate border zone, NCCs undergo epithelial-to-mesenchymal transition (EMT), delaminate, and migrate to populate their terminal sites in the developing embryo.^35^^,^^36^^,^^37^

Distinctively, NCCs are multipotent and will form characteristic ectodermal derivatives, such as peripheral neurons, as well as form cell types that are typically mesodermal derived such as cardiac tissue and craniofacial bone and cartilage.^38^ Cranial neural crest cell (CNCC)-derived bone and cartilage form the frontal, nasal, zygomatic, maxillary, mandibular, and dentary bones, as well as the bones of the inner ear and the hyoid.^37^^,^^39^ Moreover, malformations of these bones underlie many of the craniofacial anomalies seen in individuals with CSS, including a depressed nasal bridge, short nose, averted nares, broad philtrum, and wide mouth.^32^ Similarly, *Arid1a* conditional knockout models in the neural crest of developing mouse embryos^40^ recapitulate these craniofacial phenotypes that are characteristic of CSS. Together, the clinical manifestations of individuals with *ARID1A*-CSS and the craniofacial anomalies in conditional *Arid1a*^*+/−*^ mouse models demonstrate the indispensable role of ARID1A-BAF in neural crest formation and function. However, the molecular pathways regulated by ARID1A during craniofacial development and CNCC specification remain poorly understood.

Herein, we sought to uncover the role of ARID1A-BAF in the specification and migration of CNCCs in the context of craniofacial development. Our data suggests that ARID1A-BAF is required to activate gene networks associated with EMT. Using induced pluripotent stem cells (iPSCs) derived from individuals with *ARID1A*^+/−^ CSS, we discovered impaired *ARID1A* correlates with the upregulation of neuronal networks at the expense of EMT networks. Motifs matching the binding site for the ZIC2 transcription factor were highly enriched at the ARID1A-bound genes linked to EMT, and *in vitro* and *in vivo* experiments suggest ARID1A-mediated induction of EMT occurs via ZIC2. These findings highlight an essential axis involving an ARID1A-ZIC2 interaction required for NCC EMT and suggests a pathogenic mechanism that may underlie the *ARID1A*-associated CSS phenotypes.

## Material and methods

### Human iPSC collection, reprogramming, and culture

The CTRL1 iPSC line was obtained from the iPSC Core at the University of Pennsylvania (SV20, male, age 43). Skin fibroblasts were obtained from two pediatric individuals with CSS (CSS1, teenage male; CSS2, female) by the team of Dr. Gijs Santen at Leiden University (LUMC).

We complied with all relevant ethical regulations for work with human participants and informed consent was obtained by LUMC, which approved the protocol under the coordination of Dr. Gijs Santen. The study was conducted in accordance with the criteria set by the Declaration of Helsinki. The protocol to make iPSCs was approved by the institutional review board (IRB) at LUMC. Collecting patient-derived material and establishing iPSCs were all performed according to local (LUMC) IRB protocols.

The fibroblasts were reprogrammed into iPSCs with the polycistronic lentiviral vector LV.RRL.PPT.SF.hOKSM.idTomato.-preFRT^41^ as described elsewhere.^42^ Multiple clones per line were derived and one *ARID1A* mutant clone per line was used for this study: CSS1 = LUMC0081iARID02 or 81-2 and CSS2 = LUMC0064iARID04 or 64-4.

Since CSS2 exhibited somatic mosaicism, *ARID1A* wild-type iPSC clones were also isolated from CSS2 (CTRL2; LUMC0064iARID09 or 64-9). For each clone per line, pluripotency was assessed by immunofluorescence microscopy using antibodies against NANOG, OCT3/4, SSEA4, and Tra-1-81 under maintenance conditions and antibodies against TUBB3, AFP, and CD31 after spontaneous differentiation into the three germ layers as described elsewhere.^41^^,^^43^ Clones with proper pluripotent characteristics were selected for downstream usage. Karyotyping by G banding was assessed for all three lines by the LUMC, and short tandem repeat profiling was performed by the LUMC, and all four lines were profiled at the Stem Cell and Regenerative Neuroscience Center at Thomas Jefferson University. The iPSC lines were expanded in mTeSR1 medium (STEMCELL Technologies) on Matrigel (BD Pharmingen) or GeltrexTM LDEV-Free hESC-qualified Reduced Growth Factor Basement Membrane Matrix (Fisher Scientific). Small cell clusters (50–200 cells) were passaged ∼1:10 at 80% confluency using 0.5 mM EDTA (STEMCELL Technologies).

### RNA extraction followed by sequencing

Cells were lysed in Tri-reagent (Zymo) and total RNA was extracted using Direct-zol RNA Miniprep kit (Zymo) according to the manufacturer’s instructions. RNA was quantified, and RNA integrity was checked by Tapestation 4150 (Agilent). Only samples with an RNA integrity number above 8.0 were used for transcriptome analysis. RNA libraries were prepared using 1 μg of total RNA input using NEB-Next Poly(A) mRNA Magnetic Isolation Module, NEBNext UltraTM II Directional RNA Library Prep Kit for Illumina and NEBNext Multiplex Oligos Dual Index Primers for Illumina (New England BioLabs) according to the manufacturer’s instructions (New England Biolabs). Libraries were sequenced on a NextSeq2K (Illumina) generating single-end ∼138-bp reads. Each cell line at the specified timepoint(s) were collected from three separate differentiations for emphasis on reproducibility.

### RNA-sequencing analysis

All raw fastq files were analyzed via FastQC (https://github.com/s-andrews/FastQC), and adapters were removed with TrimGalore (https://github.com/FelixKrueger/TrimGalore). Kallisto^44^ was used to quantify reads mapping to each gene. Differential gene expression levels were determined using DESeq2.^45^ Briefly, abundance for each replicate of each sample at the specified timepoint were converted to both raw gene count and transcript per million (TPM). The raw gene count for each sample (including replicates) was used to determine differential gene expression with DESeq2 (*p* value < 0.05 and FDR < 5%) and were grouped based on condition (either CTRL or CSS). The TPMs were used for the violin plots comparing expression levels of individual genes. All statistical analyses were performed using the latest versions of BEDTools,^46^ deepTools,^47^ R, and Prism.

### *In vitro* immunohistochemistry

Immunohistochemistry (IHC) of iPSCs and CNCCs was performed in μ-Slide 4 Well Glass Bottom (IBIDI 80426). Upon fixation (4% formaldehyde for 10 min), cells were permeabilized in blocking solution (0.1% Triton X-100, 1× phosphate buffered saline [PBS], 5% normal donkey serum) and then incubated with the antibody of interest. The total number of cells in each field was determined by counterstaining cell nuclei with 4,6-diamidine-2-phenylindole dihydrochloride (DAPI; Sigma-Aldrich; 50 mg/mL in PBS for 15 min at room temperature). Immunostained cells were analyzed via confocal microscopy using a Nikon A1R+. Images were captured with a 4× objective for iPSCs and a 60× objective for CNCCs with a pinhole of 1.0 Airy unit. Analyses were performed in sequential scanning mode to rule out cross bleeding between channels. Fluorescence intensity and counting quantifications were performed with Fiji (https://fiji.sc) and the NIS-Elements AR software (https://www.microscope.healthcare.nikon.com/en_AOM/products/software/nis-elements/nis-elements-advanced-research). In detail, to analyze the net intensity of the cells, we created a mask around each nucleus using DAPI intensity as the base criterion. This mask served as the reference for measuring the fluorescence intensity or number of cells positive for nuclear proteins. To analyze cytoplasmic proteins, the previously drawn nucleus mask was expanded to encompass the entire cytoplasm. All antibodies are listed in Table S1. All two-tailed unpaired t tests were performed in Prism.

### CNCC specification

The iPSC lines were specified into CNCC as previously described.^3^^,^^48^ In brief, iPSCs were cultured with CNCC specification media: 1:1 Neurobasal medium/D-MEM F-12 medium (Invitrogen), 1× penicillin-streptomycin solution, 1× Glutamax supplement (100× stock, Invitrogen), 0.5× B-27 supplement with vitamin A (50× stock, Invitrogen), 0.5× N-2 supplement (100× stock, Invitrogen), 5 μg/mL bovine insulin (Sigma-Aldrich), 20 ng/mL EGF (epidermal growth factor; Sigma-Aldrich), and 20 ng/mL hFGF (human fibroblast growth factor; Biolegend) for 6 days. Medium was changed every 1–2 days. At day ∼7 when early migratory CNCCs first appeared, the cells were transitioned to CNCC early maintenance media: 1:1 Neurobasal medium/D-MEM F-12 medium (Invitrogen), 1× penicillin-streptomycin solution, 1× Glutamax supplement (100× stock, Invitrogen), 0.5× B-27 supplement with vitamin A (50× stock, Invitrogen), 0.5× N-2 supplement (100× stock, Invitrogen), 1 mg/mL bovine serum albumin, serum replacement grade (Gemini Bio-Products # 700-104 P), 20 ng/mL EGF (Sigma-Aldrich), and 20 ng/mL hFGF (Biolegend) for 7 days. After 14 full days of CNCC specification, the cells were maintained in an “early CNCC” state for up to day 21 (seven additional days) for subsequent experiments, or immediately transitioned to CNCC late maintenance media: 1:1 Neurobasal medium/D-MEM F-12 medium (Invitrogen), 1× penicillin-streptomycin solution, 1× Glutamax supplement (100× stock, Invitrogen), 0.5× B-27 supplement with vitamin A (50× stock, Invitrogen), 0.5× N-2 supplement (100× stock, Invitrogen), 1 mg/mL bovine serum albumin, serum replacement grade (Gemini Bio-Products # 700-104 P), 20 ng/mL EGF (Sigma-Aldrich), 20 ng/mL hFGF (Biolegend), 3 μM ChIRON 99021 (Selleck Chem S1263), and 50 ng/mL bone morphogenetic protein 2 (BMP2) (Peprotech 120-02). CNCCs transitioned to late maintenance media can be maintained for up to two additional weeks.

### Flow cytometry

CTRL and CSS patient-derived iPSCs were treated with accutase for 5 min to obtain a single-cell suspension. Cells were then washed with cold 1× PBS + 2% fetal bovine serum (FBS), and live cells were counted and resuspended in 1× PBS to 1 × 10^6^ cells/mL. LIVE/DEAD aqua stain (ThermoFisher L34965) was prepared following the manufacturer’s instructions, and 1 μl of prepared stain was added per 1 × 10^6^ cells and incubated for 30 min at 4°C in the dark. Cells were then washed twice in 1× PBS + 2% FBS. Cells were resuspended in up to 100 μl 1× PBS +2% FBS and 2 μl of respective antibodies (1:50 dilution; Table S1) and incubated for 15 min at 4°C in the dark. The antibodies were removed, and the cells were washed once in 1× PBS + 2% FBS. Stained cells were resuspended in 300 μl of PBS + 2% FBS and strained through a 35 μM strainer. Flow cytometry data were acquired using a BD Celesta and analyzed with FlowJo Software v10.10.

### Western blotting

Cells were washed three times in 1× PBS and lysed in radioimmunoprecipitation assay buffer (RIPA buffer; 50 mM Tris-HCl pH 7.5, 150 mM NaCl, 1% Igepal, 0.5% sodium deoxycholate, 0.1% sodium dodecyl sulfate [SDS] 500 μM DTT) with protease inhibitors. Whole-cell lysate was loaded in Novex WedgeWell 4%–20% Tris-Glycine Gel (Invitrogen) and subject to SDS-PAGE. Proteins were then transferred to an Immun-Blot polyvinylidene fluoride or Nitrocellulose membrane (Thermo Fisher) for antibody probing. Membranes were blocked in a 10% BSA/Tris-buffered saline with Tween (TBST) solution for 30 min then incubated with diluted primary antibodies (Table S1) in a 5% BSA/TBST solution. Membranes were then washed with TBST and incubated with diluted secondary antibodies (Table S1). Chemiluminescent signal was detected using the KwikQuant Western Blot Detection Kit (Kindle BioSciences) or the Amersham ECL Prime Western Blotting Detection Reagents (Cytiva) and a KwikQuant Imager.

### Transwell assay

The transwell assay was performed using the Cell Migration Assay Kit (abcam ab235694) following the manufacturer’s protocol. Briefly, CNCCs were cultured to ∼80% confluency and growth factors (EGF and FGF) were removed from the CNCC early maintenance media for 24 h to prevent proliferation. The bottom chamber of the migration plate was prepared with growth-factor-free CNCC early media and 10% FBS, and the top chamber was assembled. Cells were harvested and counted, and 200,000 cells were resuspended in growth-factor-free CNCC early maintenance media and seeded in the top chamber. Cells were incubated in the migration chamber at 37°C for 48 h. After 48 h, the cells that invaded through the migration membrane were dissociated and incubated in cell dye for 1 h at 37°C. One set of standards per cell type was created via serial dilution and incubated in cell dye for 1 h at 37°C. Absorbance for each sample and standard was read at Ex/Em-530/590 nm using a PolarStar Optima plate reader (BMG LabTech). Percent migration was calculated from the linear curve of the plotted standards.

### Scratch wound assay

CNCCs were cultured to ∼80% confluency, and growth factors (EGF and FGF) were removed from the CNCC early maintenance media for 24 h to prevent proliferation. Cells were harvested and counted, and 100,000 cells were resuspended in growth-factor-free CNCC early maintenance media and seeded in an IncuCyte ImageLock 96-well plate (Sartorius 4379) in triplicate and incubated for 48 h at 37°C. The IncuCyte 96-Well Woundmaker Tool was used to create identical wounds in each well. The wells were washed twice to remove the cells dislodged from the Woundmaker Tool, and the media was refreshed. The cells were incubated at 37°C, and migration was assayed for 96 h with repeat scanning every 2 h via the IncuCyte ZOOM Live-Cell Analysis System. Average percent confluence and wound density was quantified and normalized relative to each individual cell count per replicate.

### ChIP sequencing

All samples from different conditions were processed together to prevent batch effects. Approximately 13 million cells were cross-linked with 1% formaldehyde for 5 min at room temperature, quenched with 125 mM glycine, harvested, and washed twice with 1× PBS. The fixed cell pellet was resuspended in chromatin immunoprecipitation (ChIP) lysis buffer (150 mM NaCl, 1% Triton X-100, 0.7% SDS, 500 μM DTT, 10 mM Tris-HCl, 5 mM EDTA), and chromatin was sheared to an average length of 200–900 base pairs using a Covaris S220 Ultrasonicator at 5% duty factor between 6 and 9 min. The chromatin lysate was diluted with SDS-free ChIP lysis buffer. 15 μg of antibody was used for ARID1A and ZIC2 and 3 μg of antibody for H3K27ac (Table S1). The antibody was added to at least 5 μg of sonicated chromatin along with Dynabeads Protein G magnetic beads (Invitrogen) and incubated with rotation at 4°C overnight. The beads were washed twice with each of the following buffers: Mixed Micelle Buffer (150 mM NaCl, 1% Triton X-100, 0.2% SDS, 20 mM Tris-HCl, 5 mM EDTA, 65% sucrose), Buffer 200 (200 mM NaCl, 1% Triton X-100, 0.1% sodium deoxycholate, 25 mM HEPES, 10 mM Tris-HCl, 1 mM EDTA), LiCl detergent wash (250 mM LiCl, 0.5% sodium deoxycholate, 0.5% NP-40, 10 mM Tris-HCl, 1 mM EDTA), and a final wash was performed with cold 0.1× Tris-EDTA (TE). Finally, beads were resuspended in 1× TE containing 1% SDS and incubated at 65°C for 10 min to elute immunocomplexes. The elution was repeated twice, and the samples were incubated overnight at 65°C to reverse cross linking along with the input (5% of the starting material). The DNA was digested with 0.5 mg/mL proteinase K for 1 h at 65°C and then purified using the ChIP DNA Clean & Concentrator kit (Zymo) and quantified with QUBIT. Barcoded libraries were made with the NEBNext Ultra II DNA Library Prep Kit for Illumina using NEBNext Multiplex Oligos Dual Index Primers for Illumina (New England BioLabs) and sequenced on NextSeq2K (Illumina) producing ∼138 bp single-end reads.

### ATAC sequencing

For assay for transposase-accessible chromatin with sequencing (ATAC-seq) experiments, 50,000 cells per condition were processed as previously described.^49^ Briefly, 50,000 cells were collected, washed, and lysed. The chromatin was subjected to transposition/library preparation via a Tn5 transposase using the Tagment DNA Enzyme and Buffer Kit (Ilumina 20034197) and incubated at 37°C for 30 min with slight agitation. Transposed DNA was purified using a MinElute PCR Purification Kit (Qiagen 28004). Transposed DNA fragments were then amplified using a universal and barcoded primer.^49^ Thermal cycling parameters were set as follows: 1 cycle of 72°C for 5 min and 98°C for 30 s, followed by 5 cycles of 98°C for 10 s, 63°C for 30 s, and 72°C for 1 min. The amplification was paused, and 5 μL of the partially amplified, transposed DNA was used for a qPCR side reaction, including the universal and sample-specific barcoded primers,^49^ PowerUp SYBR Green Master Mix (Applied Biosystems), NEBNext High-Fidelity 2× PCR Master Mix, and nuclease-free water. The qPCR side reaction parameters were set as follows: 1 cycle of 72°C for 5 min and 98°C for 30 s, followed by 40 cycles of 98°C for 10 s, 63°C for 30 s, and 72°C for 1 min. The Rn vs. cycle was plotted to determine the remaining number of PCR cycles needed where 1/3 of the maximum fluorescent intensity corresponds to the additional cycle number. The remaining partially amplified transposed DNA was fully amplified using the previous parameters with the additional cycle number determined from the qPCR side reaction. The amplified, transposed DNA was purified using AMPure XP beads (Beckman Coulter A63881) and sequenced using an Illumina NextSeq2K, generating ∼138 bp single-end reads.

### ChIP- and ATAC-seq analysis

After removing the adapters with TrimGalore, the sequences were aligned to the human reference genome hg19 using the Burrows-Wheeler Alignment tool with the MEM algorithm.^50^ Aligned reads were filtered based on mapping quality (MAPQ > 10) to restrict our analysis to higher quality and uniquely mapped reads, and PCR duplicates were removed. For ATAC-seq analysis, all mapped reads were offset by +4 bp for the forward strand and −5 bp for the reverse strand. MACS2^51^ was used to call peaks using the default parameters at 5% FDR. All statistical analyses were performed using the latest versions of BEDTools,^46^ deepTools,^47^ R, and Prism. Peaks were visualized in Integrative Genomics Viewer (https://igv.org) on genome build hg19.

### Motif analysis

Fasta files for the coordinates of interest were produced using BEDTools.^46^ Motif analysis of all lost ARID1A-bound regions, ARID1A-bound promoters, ARID1A “gained” regions, and ZIC2 relocated regions was performed using HOMER.^52^ Shuffled input sequences were used as background. E values <0.001 were used as a threshold for significance. Transcription factor motif quantification was performed using FIMO^53^ (The MEME Suite) on a background of lost ARID1A-bound regions.

### Mouse lines

The B6;FVB-Tg(*Zic2*-EGFP)HT146Gsat/Mmucd line (identification number RP23-158G6)^54^ was generated by GENSAT^55^ and obtained from the Mutant Mouse Regional Resource Center (https://www.mmrrc.org/catalog/sds.php?mmrrc_id=30037). Hereafter, this line is known as Tg(*Zic2*^EGFP^). The B6.129S4-*Zic2*^tm1Jaru^/JaruRbrc strain (Mouse Genome Informatics: 2156825; RBRC00165)^56^ was obtained from the RIKEN repository. Hereafter, this line is known as *Zic2*^kd^. *Zic2*^+/+^;Tg(*Zic2*^EGFP^) and *Zic2*^kd/kd^;Tg(*Zic2*^EGFP^) embryos were obtained from breeding *Zic2*^+/kd^;Tg(*Zic2*^EGFP^) mice and are referred to as *Zic2*-WT and *Zic2*-mutant, respectively. The day a vaginal plug appeared was considered embryonic day E0.5. All mouse lines were congenic on a C57BL/6J background and were kept in a timed pregnancy-breeding colony at the Instituto de Neurociencias. The animal protocols were approved by the Institutional Animal Care and Use Committee and met European and Spanish regulations.

### Constructs and *in vivo* electroporation

Fertilized White Leghorn chicken eggs were incubated at 38°C until the desired developmental stage, according to Hamburger and Hamilton.^57^ Platinum electrodes were used with 5× 10 ms pulses at 25 V generated with a TSS20 Ovodyne electroporator. The following plasmids were injected at the indicated concentrations: pCAG-*ZIC2* plasmid (1 μg/μL) and CAG-GFP (0.5 μg/μL). Due to technicalities underlying electroporation, all analyses of electroporated chicken embryos were confined to the trunk region.

### *In vivo* IHC and iDISCO

For IHC, chick embryos fixed overnight at 4°C, and mouse embryos fixed for 4–5 h (E8.5–E9.5) with 4% paraformaldehyde (PFA)/PBS and were washed with PBS. Cryostat (40 μm) and vibratome (70 μm) sections or whole embryos were incubated with the corresponding primary and secondary antibodies, according to standard protocols. For immunolabeling-enabled three-dimensional imaging of solvent-cleared organs (iDISCO), embryos were fixed overnight at 4°C with 4% PFA/PBS, and immunolabeling was performed before clarification. The iDISCO protocol was performed as previously published.^58^ IHC images were captured with an Olympus FV1000 confocal IX81 microscope/FV10-ASW Software. Images of iDISCO-clarified embryos were obtained with an Ultramicroscope II (LaVisionBiotec). All antibodies are listed in Table S1.

### *In vivo* IHC analysis

The number of Sox10^+^ or FoxD3^+^ cells per slice was quantified and normalized by neural tube length and the ratio of positive cells inside the tube compared with the total number of cells in each cryosection (30 μm). A region of interest was delineated in the migration zone outside the neural tube, and the fluorescence intensity was measured (IntDen parameter in Fiji/ImageJ) and normalized to total fluorescence. Two complementary regions of interest were delineated along the migratory stream (one in the ventromedial area and another in the dorsolateral area). GFP fluorescence intensity was measured, and the total fluorescence area was calculated as integrated density (fluorescence of the selected area − mean background fluorescence) in Fiji. All two-tailed unpaired t tests were performed in Prism.

### Statistical and genomic analysis

All statistical analyses were performed using the latest versions of BEDTools,^46^ deepTools,^47^ R, and Prism. Superplot quantification represents at least *n* = 3 represented by a distinctive shape with each small data point representing a captured image. The larger data points correspond to the average values of each replicate. A two-tailed unpaired t test performed on the average values. A value of *p* < 0.05 was considered significant; ^∗^*p* < 0.05; ^∗∗^*p* < 0.01; ^∗∗∗^*p* < 0.001; ^∗∗∗∗^*p* < 0.0001; and n.s., not significant. Error bars represent standard error of the mean. Pathway analysis was performed with Ingenuity-Pathway Analysis Suite (IPA; QIAGEN Inc., https://digitalinsights.qiagen.com/IPA).

## Results

### Control and CSS patient-derived iPSCs are pluripotent

**Figure 1:**
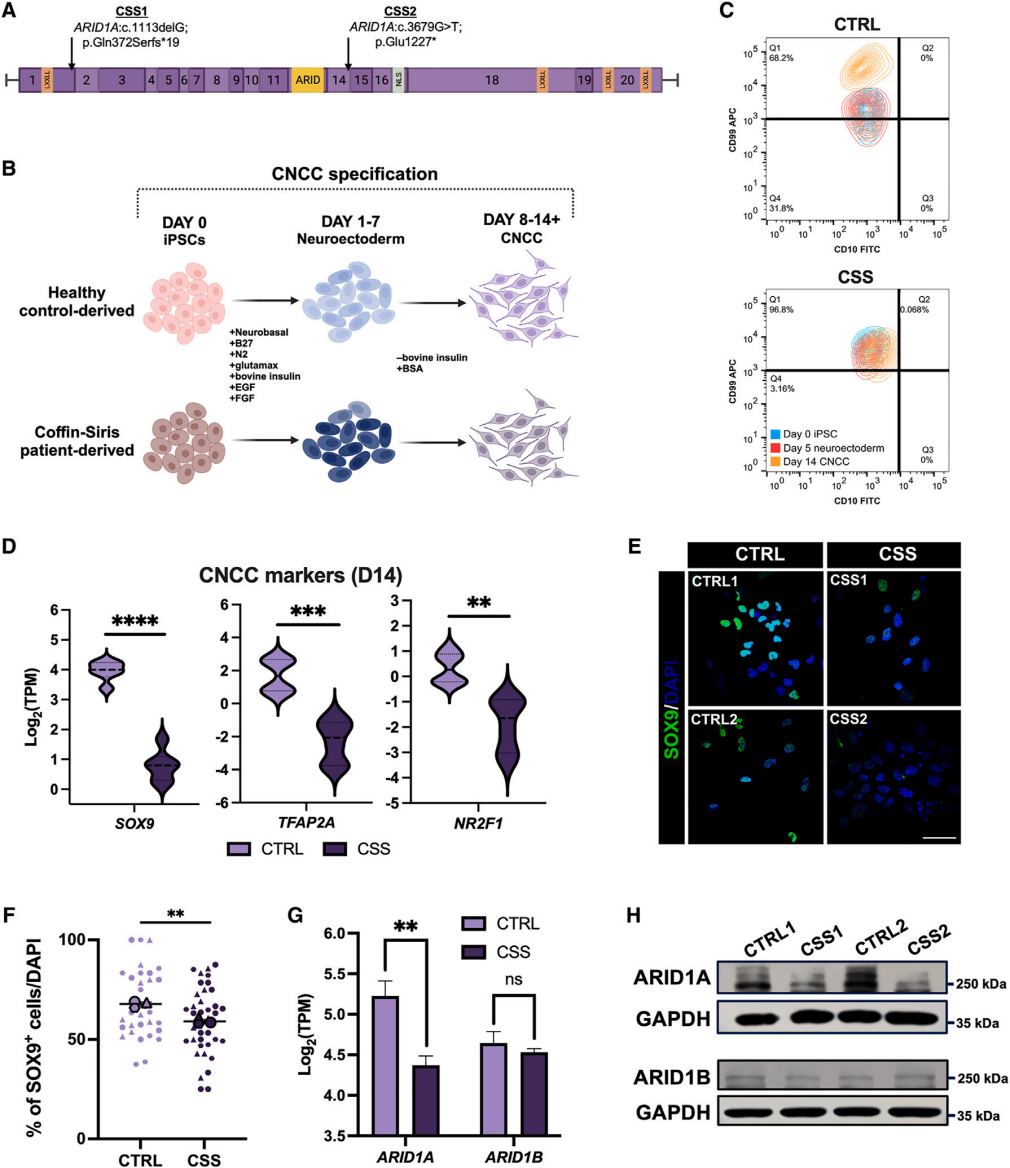
*ARID1A*-haploinsufficient cells fail to specify as CNCCs (A) Schematic of the *ARID1A* gene (GenBank: NM_006015.6) highlighting the two nonsense mutations in each CSS cell line. Each number represents a corresponding exon in *ARID1A*. LXXLL, protein-interaction domain; ARID, AT-rich interaction domain or DNA-binding domain; NLS, nuclear localization signal. Made with BioRender.com. (B) Graphical illustration of 2D *in vitro* CNCC specification. EGF, epidermal growth factor; FGF, fibroblast growth factor ; BSA, bovine serum albumin. Made with BioRender.com. (C) CSS lines at day 14 (D14) fail to show a distinctive increase in the CD99 surface marker representative of successful CNCC specification. All populations (D0 iPSC, D5 neuroectoderm, D14 CNCC) are CD10^+^ with a slight increase in the CD10 surface marker at D14, which is characteristic of preparation of a differentiation trajectory toward the mesenchymal lineage. (D) Violin plots displaying log_2_(TPM) of CNCC markers *SOX9*, *TFAP2A*, and *NR2F1* at D14 between CTRL and CSS cell lines. A two-tailed unpaired t test was performed and *p* < 0.05 was considered significant; *SOX9*: ^∗∗∗∗^*p* < 0.0001, *TFAP2A*: ^∗∗∗^*p* = 0.0001, and *NR2F1*: ^∗∗^*p* = 0.0012. (E and F) (E) Representative images and (F) quantification of a SOX9 immunofluorescence performed at D14 in CTRL and CSS cell lines. (E) DAPI staining on nuclei in blue. Merged images shown taken at 60× magnification; scale bar, 50 μm. (F) SuperPlot quantification of percentage of SOX9^+^ cells per DAPI. *n* = 3 represented by a distinctive shape with each small data point representing a captured image. The larger data points correspond to the average values of each replicate. A two-tailed unpaired t test performed on the average values between CTRL and CSS cell lines; *p* < 0.05 was considered significant; ^∗∗^*p* = 0.0032. (G and H) (G) Log_2_(TPM) bar plot and (H) immunoblot of ARID1A and ARID1B at day 10 of CNCC specification between CTRL and CSS cell lines. Reduction of *ARID1A* transcript and protein levels correspond to the *ARID1A*-haploinsufficient genotype while ARID1B levels are consistent across CTRL and CSS cell lines. A two-tailed unpaired t-test was performed in (G) and *p* < 0.05 was considered significant; *ARID1A*: ^∗∗^*p* = 0.0029, *ARID1B*: ns *p* = 0.4592. Error bars represent the standard error of the mean.

We leveraged control and *ARID1A* variant-containing, human-derived iPSCs, to investigate the role of ARID1A in the BAF chromatin remodeling complex during cranial neural crest specification and craniofacial development. To generate these iPSCs, skin fibroblasts were collected from two individuals with CSS each harboring distinct, previously reported heterozygous, *de novo*, nonsense mutations in *ARID1A*^17^ (MIM: 614607). CSS patient 1 -derived cells (CSS1) originated from a teenage male harboring a *ARID1A:*c.1113delG (p.Gln372Serfs^∗^19) variant and CSS patient 2-derived cells (CSS2) originated from a female harboring a *ARID1A:*c.3679G>T (p.Glu1227^∗^) variant (Figure 1A; GenBank: NM_006015.6). Both variants were classified as pathogenic based on the criteria set forth by the American College of Medical Genetics and Genomics.^59^ A subsequent *ARID1A* wild-type iPSC line was used as a control (CTRL1). While germline mutations in *ARID1A* lead to a severe CSS subtype with multiple congenital anomalies and reduced survival, mosaic variants are often observed in individuals with a more moderate CSS phenotype.^60^ The *ARID1A*-haploinsufficient CSS fibroblasts (CSS1 and CSS2) were reprogrammed into iPSCs (Figure S1A), and due to the associated mosaicism, it was possible to isolate *ARID1A* wild-type iPSCs (CTRL2) in addition to the *ARID1A*-haploinsufficient iPSCs from CSS2 for an isogenic study system. Genotypes of the resulting iPSC lines were verified via deep sequencing.

Previous studies from our lab^3^ and others^61^^,^^62^ have shown that ARID1A-containing BAF is present in pluripotent stem cells (esBAF; Figure S1B). Additionally, GBAF (non-canonical BAF) is an alternate embryonic stem cell-specific configuration of BAF that lacks ARID subunits and instead incorporates GLTSCR1/GLTSCR1L and BRD9.^5^^,^^13^ Notably, GBAF is also present in embryonic stem cells where it is implicated in regulating pluripotency.^5^ Consistent with the lack of ARID subunits in GBAF, the *ARID1A*-haploinsufficient iPSCs display regular iPSC morphology, including small cells making up distinct, circular, compact colonies with well-defined colony borders (Figure S1C) and broadly express OCT4, SOX2, and NANOG mRNA with comparable protein levels (Figures S1D–S1F). RNA-seq of iPSCs identified only 121 differentially expressed genes between CTRL and CSS cells (*p* < 0.05; FDR < 5%; log_2_(fold change)+/−1; Table S2). Notably, genes involved in pluripotency regulation such as *OCT4*, *SOX2*, and *NANOG* were not differentially expressed (Table S2). These data show that CSS patient-derived iPSCs maintain pluripotency and *ARID1A*-haploinsufficiency did not impact pluripotent gene expression in this cell type.

### CSS cranial NCCs exhibit migratory defects

As premature loss of pluripotency did not appear to be the pathological mechanism of *ARID1A* haploinsufficiency, we next investigated the role of ARID1A in cranial neural crest specification. CTRL and CSS iPSCs were specified to CNCCs as per previously published protocols^48^^,^^63^^,^^64^ (Figure 1B). RNA-sequencing analysis comparing CTRL iPSCs (Table S3) and derived CNCCs (Table S4) revealed a transcriptional signature in which CNCC markers were highly expressed while pluripotency genes were downregulated (Figure S1G), supporting successful CNCC specification (Figure S1H).

Previous work from our group demonstrated a bimodal switch between ARID1A and ARID1B in the BAF complex during the CNCC specification process^3^ (Figure S1B). At the onset of neuroectoderm formation, ARID1A protein levels are downregulated and nearly undetectable while ARID1B protein levels increase^3^ (Figure S1B). ARID1B levels remain elevated throughout the duration of the neuroectodermal stage (days 1–7) but are then downregulated during the onset of EMT and CNCC formation. At this stage, ARID1A protein levels are re-upregulated. This latter switch occurs at day 8–9 of our CNCC specification protocol, and ARID1A is retained in the BAF complex as migratory CNCCs are formed^3^ (Figure S1B). Notably, this time point immediately precedes EMT and delamination of the CNCCs as they acquire a migratory phenotype.

To investigate the impact *ARID1A* haploinsufficiency has on CNCC identity, we harvested specified CNCCs on day 14. At this stage, CTRL cells displayed characteristic single-cell CNCC morphology, while CSS patient-derived cells adopted a more elongated and clustered cellular morphology (Figure S1I). By day 14, CSS cell lines failed to form the distinct CD99^+^ CNCC population, which is characteristic of successful specification (Figures 1C and S2) as observed by flow cytometry. Contrary to results observed in iPSCs, RNA sequencing performed in CTRL and CSS-derived CNCCs at day 14 revealed over 3,800 differentially expressed genes (*p* < 0.05; FDR <5%; log_2_(fold change) +/−1; Table S5). Notably, genes associated with CNCC specification and migration (i.e., *SOX9*, *TFAP2A*, and *NR2F1*) were significantly decreased in the CSS cells relative to CTRL CNCCs (Figure 1D). Immunostaining at day 14 validated the transcriptomic data showing a reduced number of SOX9^+^ CSS cells relative to CTRLs (Figures 1E and 1F).

As ARID1A levels are upregulated by day 8–9 of our *in vitro* CNCC specification program,^3^ we chose day 10 for comprehensive genomic studies, focused on the role of ARID1A during CNCC formation. First, we wanted to elucidate if other ARID proteins (i.e., ARID1B or the PBAF member ARID2) could compensate for *ARID1A* haploinsufficiency. As expected, ARID1A protein levels were significantly diminished in the CSS lines at day 10 of CNCC formation (Figures 1G and 1H). However, neither *ARID1B* nor *ARID2* were upregulated at the transcript (Figures 1G and S1J) or protein level (Figure 1H) at the same time point, suggesting other BAF configurations could not compensate for the partial loss of *ARID1A* during CNCC formation. Together, these data implicated ARID1A-BAF as a key regulator of CNCC formation.

### *ARID1A*-haploinsufficiency results in decreased expression of EMT genes and increased expression of neuronal genes

**Figure 2:**
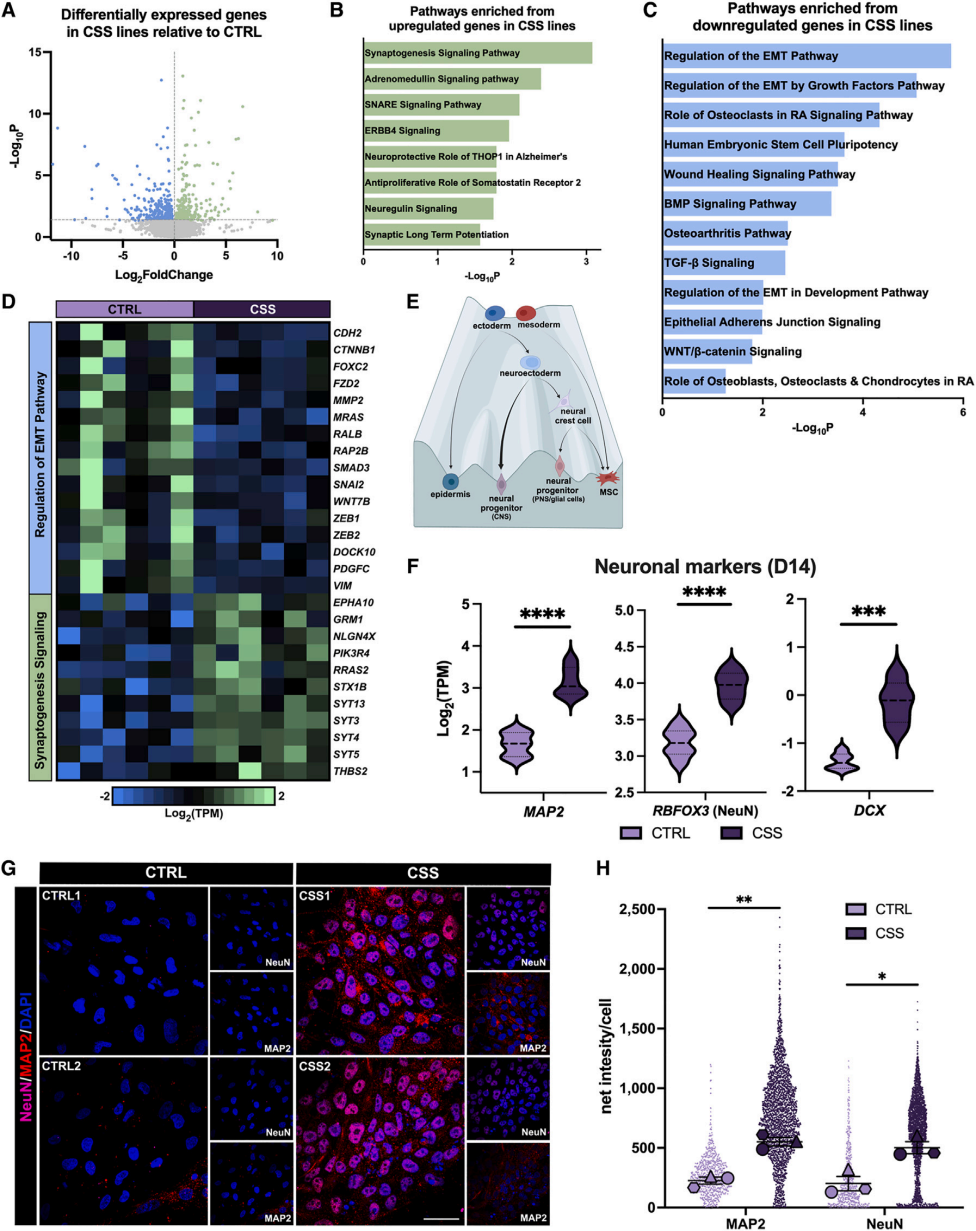
CSS patient-derived cell lines do not undergo EMT and default to the neuronal lineage (A) Volcano plot of differentially expressed genes in CSS cell lines relative to CTRLs at day 10 of CNCC specification as determined by DESeq2.^45^ Green points represent upregulated genes while blue points represent downregulated genes. *n* = 652. (B) Neuronal pathways are enriched from differentially upregulated genes in CSS cell lines at day 10 of CNCC specification and the respective −log_10_*p* value as determined by IPA. (C) Pathways enriched from differentially downregulated genes in CSS cell lines at day 10 of CNCC specification and the respective −log_10_*p* value as determined by IPA. Identified downregulated pathways are essential for successful CNCC specification, including epithelial-to-mesenchymal transition (EMT), BMP, TGF-β, and Wnt signaling. RA, rheumatoid arthritis. (D) Heatmap of genes involved in the most significant upregulated and downregulated pathways presented in (B) and (C) between CTRL and CSS cell lines. Genes involved in the Regulation of EMT pathway were downregulated while there was an increase in expression of genes involved in synaptogenesis signaling pathway in CSS cell lines. CTRL and CSS columns represent three individual replicates from three separate CNCC specifications for CTRL1/CTRL2 and CSS1/CSS2, respectively. (E) Adapted schematic of Waddington’s epigenetic landscape^65^ depicting restrictive cell fate commitment in the context of neural crest formation. The mesoderm gives rise to MSC (mesenchymal stem cells), and the ectoderm differentiates into the epidermis and neuroectoderm. Neuroectodermal fate defaults to the neural progenitor lineage of the central nervous system (CNS) represented by a thick arrow when neural crest fate cannot be specified. Neuroectoderm also gives rise to multipotent NCCs, which will form various derivatives, including the craniofacial bones and cartilage and neural progenitors and glia of the peripheral nervous system (PNS). Other derivatives of these cell types are not shown in the schematic. Made with BioRender.com. (F) Violin plots displaying log_2_(TPM) of neuronal markers *MAP2*, *RBFOX3* (NeuN), and *DCX* at day 14 between CTRL and CSS cell lines. A two-tailed unpaired t test was performed, and *p* < 0.05 was considered significant; *MAP2*: ^∗∗∗∗^*p* < 0.0001, *RBFOX3* (NeuN): ^∗∗∗∗^*p* < 0.0001, and *DCX*: ^∗∗∗^*p* = 0.0001. (G and H) (G) Representative images and (H) quantification of MAP2 and NeuN immunofluorescence performed at day 14 in CTRL and CSS cell lines. (G) DAPI staining on nuclei in blue. Images taken at 60× magnification; scale bar, 50 μm. (H) SuperPlot quantification of net intensity of MAP2 and NeuN per cell. *n* = 3 represented by a distinctive shape with each small data point representing a captured image. The larger data points correspond to the average values of each replicate. A two-tailed unpaired t test performed on the average values between CTRL and CSS cell lines; *p* < 0.05 was considered significant; MAP2 ^∗∗^*p* = 0.0021 NeuN ^∗^*p* = 0.0184. Error bars represent the standard error of the mean.

In addition to the 3,800 genes differentially expressed at the end of the CNCC specification protocol (day 14; Table S5), RNA-sequencing analyses performed at day 10 of CNCC specification (i.e., at the onset of ARID1A re-upregulation) identified 652 differentially expressed genes in the CSS lines compared to CTRLs (Figure 2A; Table S6). Of these 652 genes, 349 were downregulated and 303 were upregulated in CSS lines. Downregulated genes were significantly enriched in pathways associated with EMT while the upregulated genes were significantly enriched in neuronal pathways (Figures 2B–2D). Notably, both neural progenitors and NCCs are derived from the neuroectoderm (Figure 2E). We previously demonstrated that this developmental precursor was regulated by a specific version of BAF incorporating ARID1B rather than ARID1A.^3^ However, the *ARID1A*-haploinsufficient lines failed to upregulate EMT and CNCC genes while aberrantly upregulating neuronal genes. Thus, the partial loss of *ARID1A* in CSS cells appeared to impair formation of the multipotent CNCC lineage from neuroectoderm, triggering a default differentiation to the neuronal lineage (Figure 2E).

Further, we examined transcript levels of neuronal markers at the conclusion of the CNCC specification process (day 14). The expression of neuronal genes *MAP2*, *RBFOX3* (NeuN), and *DCX* were significantly upregulated in CSS lines compared to CTRLs (Figure 2F). Moreover, immunofluorescence for MAP2 and NeuN revealed a massive increase in both markers in the CSS cell lines (Figures 2G and 2H). Notably, these cells featured a level of heterogeneity correlated with the observed cellular morphology (Figure S1I). CSS cells that displayed a clustered and elongated morphology (Figure S1I) were positive for these neuronal markers (Figures 2G and 2H). Few isolated single cells formed in the CSS lines by day 14; however, these cells did not exhibit this aberrant neuronal protein accumulation by immunofluorescence. These data suggest that the *ARID1A*-haploinsufficient lines were unable to commit toward the CNCC lineage, while aberrantly upregulating a mix of neural progenitor and neuronal markers.

### Functional EMT is impaired in *ARID1A*-haploinsufficient CNCCs

CNCCs are a multipotent population with the potential to differentiate into both ectodermal and mesenchymal derivatives^38^ (Figure 2E). To migrate and differentiate into the terminal derivatives, CNCCs must undergo EMT allowing for delamination and migration from the neural tube.^35^^,^^36^^,^^37^ Key mechanisms involved in developmental EMT include regulation of cadherin switching whereby E-cadherin is downregulated, and N-cadherin is upregulated.^66^^,^^67^^,^^68^ This switch is accompanied by increased levels of essential mesenchymal factors vimentin, SNAIL, SLUG, and the TWIST, as well as ZEB transcription factor families.^66^

**Figure 3:**
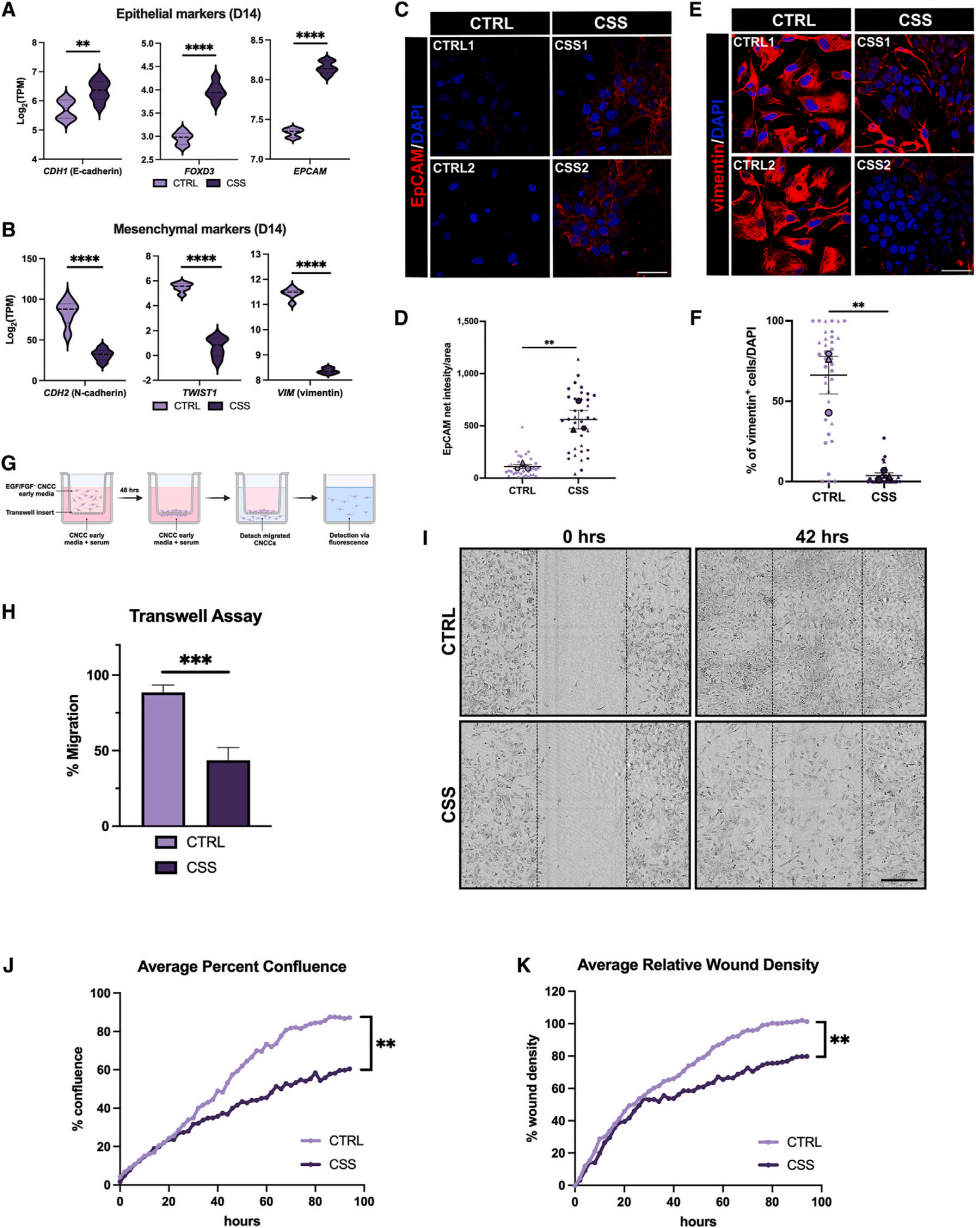
Impaired EMT compromises the specification of *ARID1A*-haploinsufficient cells into migratory CNCCs (A) Violin plots displaying log_2_(TPM) of epithelial/pre-migratory CNCC markers *CDH1* (E-cadherin), *FOXD3*, and *EPCAM* at day 14 between CTRL and CSS cell lines. A two-tailed unpaired t test was performed and *p* < 0.05 was considered significant; *CDH1* (E-cadherin): ^∗∗^*p* = 0.0079, *FOXD3*: ^∗∗∗∗^*p* < 0.0001, and *EPCAM*: ^∗∗∗∗^*p* < 0.0001. (B) Violin plots displaying log_2_(TPM) of mesenchymal/migratory CNCC markers *CDH2* (N-cadherin), *TWIST1*, and *VIM* (vimentin) at day 14 between CTRL and CSS cell lines. A two-tailed unpaired t test was performed and *p* < 0.05 was considered significant; *CDH2* (N-cadherin): ^∗∗∗∗^*p* < 0.0001, *TWIST1*: ^∗∗∗∗^*p* < 0.0001, and *VIM* (vimentin): ^∗∗∗∗^*p* < 0.0001. (C and D) (C) Representative images and (D) quantification of an EpCAM immunofluorescence performed at day 14 of CNCC specification in CTRL and CSS cell lines. (C) DAPI staining on nuclei in blue. Merged images shown taken at 60× magnification; scale bar, 50μm. (D) SuperPlot quantification of EpCAM net intensity per area. *n* = 3 represented by a distinctive shape with each small data point representing a captured image. The larger data points correspond to the average values of each replicate. A two-tailed unpaired t-test performed on the average values between CTRL and CSS cell lines; *p* < 0.05 was considered significant; ^∗∗^*p* = 0.0078. Error bars represent the standard error of the mean. (E and F) (E) Representative images and (F) quantification of a vimentin immunofluorescence performed at D14 of CNCC specification in CTRL and CSS cell lines. (E) DAPI staining on nuclei in blue. Merged images taken at 60× magnification; scale bar, 50μm. (F) SuperPlot quantification of percentage of vimentin-positive cells per DAPI. *n* = 3 represented by a distinctive shape with each small data point representing a captured image. The larger data points correspond to the average values of each replicate. A two-tailed unpaired t test performed on the average values between CTRL and CSS cell lines; *p* < 0.05 was considered significant; ^∗∗^*p* = 0.0062. Error bars represent the standard error of the mean. (G) Graphical illustration of a transwell assay. Made with BioRender.com. (H) Bar plot depicting average percent migration of CTRL and CSS cells through a transwell membrane. A two-tailed unpaired t test performed between CTRL and CSS cell lines; *p* < 0.05 was considered significant; ^∗∗∗^*p* = 0.0002. Error bars represent the standard error of the mean. (I) Representative brightfield images of a scratch wound assay (Incucyte) at 0 h and 42 h post-scratch between CTRL and CSS cell lines. 42 h represents the earliest time point of a visually closed wound in CTRL cells. Scale bar, 300 μm, 10× magnification. (J and K) (J) Average percent confluence of the wound area and (K) average relative wound density over 96 h between CTRL and CSS cell lines from the scratch wound assay performed in (I). A two-tailed unpaired t test performed between CTRL and CSS cell lines; *p* < 0.05 was considered significant; percent confluence: ^∗∗^*p* = 0.0014; percent wound density: ^∗∗^*p* = 0.0077.

Notably, we found that the expression of genes defining epithelial/neuroectodermal cells, including *CDH1* (E-cadherin) and *EPCAM*, and pre-migratory CNCCs (*FOXD3*), were all upregulated in the *ARID1A*-haploinsufficient lines compared to CTRL CNCCs (day 14; Figure 3A). Conversely, the expression of mesenchymal and migratory CNCC genes, such as *CDH2* (N-cadherin), *TWIST1*, and *VIM* (vimentin), were markedly downregulated in the *ARID1A*-haploinsufficient lines compared to CTRL CNCCs (Figure 3B). Immunostaining for the epithelial marker EpCAM (Figures 3C and 3D) and the mesenchymal marker vimentin (Figures 3E and 3F) further supported the transcriptomic data, strongly indicating that the CSS cell lines remained in a neuroepithelial state with some degree of heterogeneity (Figure 3E; CSS1), while the CTRL cells successfully underwent EMT.

To functionally assess the migratory potential of CTRL and CSS cell lines *in vitro*, we performed transwell (Figures 3G and 3H) and scratch wound assays (Figures 3I–3K). After CNCCs were specified, growth factors were removed from the media to allow for an accurate assessment of cell migration. CSS cells had approximately 50% reduced migration than CTRLs through the transwell membrane (Figure 3H). Similarly, the CTRL scratch wound was visually confluent while the CSS cell lines exhibited minimal migration (Figure 3I). A significant decrease in average percent confluence in the scratch wound as well as relative wound density was observed in CSS lines compared to CTRLs (Figures 3J and 3K). Collectively, these data suggest that EMT was affected in CSS lines as they failed to acquire a functional migratory phenotype.

### ARID1A-BAF regulates accessibility at EMT enhancers

**Figure 4:**
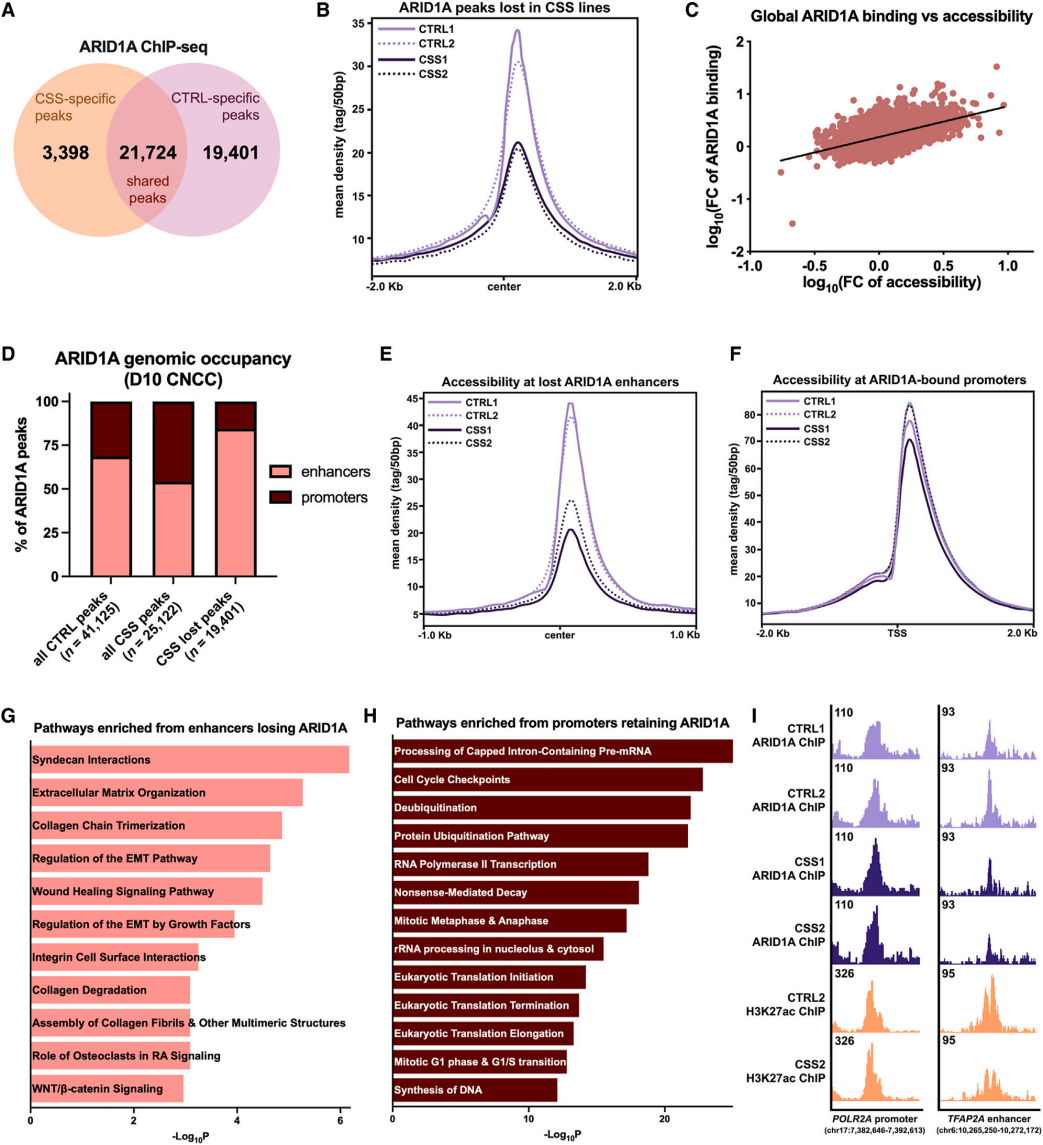
Chromatin dynamics and ARID1A genomic occupancy in CTRL and CSS cell lines (A) Venn diagram displaying the number of CSS-specific peaks (ARID1A peaks present only in CSS cell lines or “gained” in CSS cell lines; 3,398), CTRL-specific peaks (ARID1A peaks present only in CTRLs or “lost” in CSS cell lines; 19,401), and shared ARID1A peaks between CTRL and CSS cell lines (conserved ARID1A binding; 21,724) via ARID1A ChIP-seq performed at day 10 of CNCC specification. (B) Average profile of ARID1A peaks lost in CSS cell lines at day 10 (*n* = 19,401). Center represents the average overlapping global binding of ARID1A at CTRL-specific peaks. (C) Scatterplot and linear regression of the log_10_(fold-change) of ARID1A peak coverage versus the log_10_(fold-change) of ATAC peak coverage displaying a significant Pearson correlation (r = 0.4940, two-tailed ^∗∗∗∗^*p* < 0.0001) between global ARID1A binding and global accessibility. (D) Stacked bar plot depicting the percentage of ARID1A peaks enriched at enhancers (>1 kb from the closest transcription start site or TSS) and promoters (<1 kb from the closest TSS) in all CTRL ARID1A peaks (including peaks shared with CSS lines), all CSS ARID1A peaks (including peaks shared with CTRL lines), and all ARID1A peaks that are lost in CSS lines (CSS lost peaks). ARID1A peaks in CSS cell lines are enriched at promoter regions relative to CTRLs; 54% at enhancers and 46% at promoters in CSS cell lines versus 69% at enhancers and 31% at promoters in CTRLs. Approximately 85% of lost ARID1A peaks in the CSS lines were lost at enhancer regions. (E) Average profile of ATAC-seq peaks at enhancer regions that lose ARID1A binding and accessibility in the CSS cell lines at day 10 of CNCC specification (*n* = 3,203). Center represents the average overlapping lost CSS ATAC/ARID1A peaks at enhancer regions across the genome. (F) Average profile of ATAC-seq peaks at promoter regions that retain ARID1A binding and accessibility in the CSS cell lines at day 10 of CNCC specification (*n* = 11,231). TSS represents the average overlapping retained ATAC-seq peaks at ARID1A-bound transcription start sites throughout the genome. (G) Pathways enriched from genes that lose ARID1A binding at their enhancers in CSS cell lines at day 10 of CNCC specification and the respective −log_10_*p* value as determined by IPA. Pathways include those necessary for migration, extracellular matrix dynamics, and EMT. (H) Pathways enriched from genes that retain ARID1A binding at their promoters in CSS lines at day 10 of CNCC specification and the respective −log_10_*p* value as determined by IPA. Pathways include those necessary for basic cellular functions and survival including DNA synthesis, transcription, translation, and cell cycle. (I) Integrative Genomics Viewer example of a retained ARID1A peak in the CSS cell lines at the promoter of *POLR2A*, encoding the main subunit of RNA polymerase II, and a lost ARID1A peak at an enhancer for *TFAP2A*, encoding an essential CNCC marker. Representative promoter and enhancer regions marked by H3K27ac. Regions annotated using hg19.

To investigate the mechanism underlying impaired EMT in the CSS lines, we performed chromatin immunoprecipitation followed by sequencing (ChIP-seq) for ARID1A to assess alterations in binding at day 10 (i.e., ARID1A re-upregulation onset). Simultaneously, we performed an ATAC-seq to interrogate chromatin accessibility at the same time point. We detected 21,724 shared ARID1A ChIP-seq peaks across CSS and CTRL cells (Figure S3A), 3,398 new ARID1A peaks gained in CSS cell lines, and 19,401 ARID1A peaks lost in CSS lines (Figures 4A and 4B). Similarly, there were ∼46,065 conserved ATAC-seq peaks between CSS and CTRL cell lines (Figure 3SB), 9,163 peaks exclusive to CSS cells, and 20,216 peaks exclusive to CTRL cells (Figures S3C and S3D). Consistent with *ARID1A* loss being heterozygous, average profiles indicate that both ARID1A binding and chromatin accessibility at the ∼20,000 sites were nearly half of the peak height of the CTRL levels (Figures 4B and S3E). Moreover, the loss of ARID1A binding and accessibility were correlated (Figure 4C).

Analyzing the genomic occupancy of ARID1A, approximately 70% of the CTRL ARID1A ChIP-seq peaks were at enhancer regions (>1 kb from the closest transcription start site [TSS]) and 30% at promoters (<1 kb from the closest TSS; Figure 4D). Conversely, ARID1A ChIP-seq peaks in the CSS lines were enriched equally between enhancers and promoters (Figure 4D). The majority of the ARID1A peaks lost in the CSS cell lines were at enhancer regions (85% at enhancers and 15% at promoters), while most of the ARID1A peaks at promoters were retained (Figure 4D). This suggests that BAF binding at promoter regions may be essential, while BAF binding at enhancers may be dispensable. Consistent with this, accessibility and active histone marks at ARID1A-bound enhancers was significantly diminished in CSS lines (Figures 4E and S3F), while accessibility and active state at ARID1A-bound promoter regions was largely retained (Figures 4F and S3G) via ATAC-seq and H3K27ac ChIP-seq respectively. Pathway analysis on differentially expressed genes associated with enhancers that lost ARID1A binding revealed enrichment for EMT, wound healing, extracellular and collagen dynamics, and integrin interactions (Figure 4G). However, pathway analysis on genes associated with ARID1A-regulated promoters revealed enrichment for basic cellular processes including cell cycle, transcription, translation, and DNA synthesis (Figure 4H). For example, the promoter of *POLR2A*, encoding the main subunit of RNA polymerase II, harbors conserved ARID1A occupancy, while a TFAP2A enhancer displayed loss of both ARID1A binding and H3K27ac ChIP-seq signal (Figure 4I). Notably, ARID1A gained peaks were closest to aberrantly upregulated genes that were enriched for neuronal processes and cell death pathways (Figure S3H). These data suggested that ARID1A-BAF regulates EMT by opening the chromatin at EMT-associated enhancers, and the function of these enhancers is impaired in *ARID1A*-haploinsufficiency.

### ARID1A-BAF regulates the accessibility at enhancers of EMT genes to coordinate ZIC2 binding

**Figure 5:**
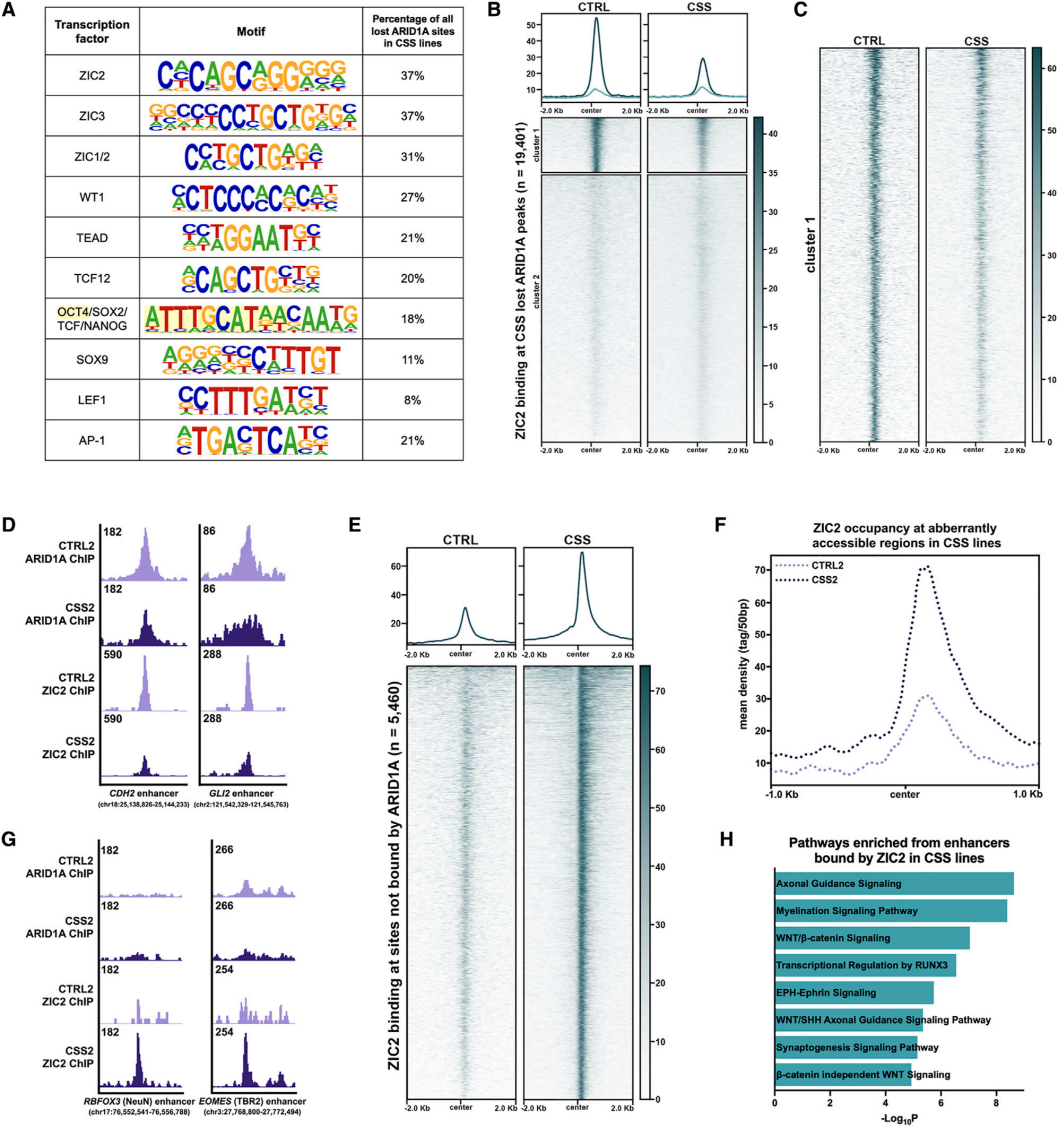
ZIC2 regulates CNCC and neuronal identity by activating accessible regulatory networks (A) Chart of enriched motifs at ARID1A-bound regions that are lost in CSS cell lines identified by HOMER. The presence of the motifs at each lost region was quantified via FIMO (MEME Suite). Yellow highlighted region represents the individual OCT4 motif. (B) Clustered heatmap of ZIC2 binding at regions that lose ARID1A binding in the CSS lines at day 10 of CNCC specification (CTRL-specific; *n* = 19,401). (C) Expanded heatmap of cluster 1 from (B). (D) Integrative Genomics Viewer example of lost ARID1A and ZIC2 peaks in CSS2 relative to the isogenic CTRL2 at ARID1A target enhancers at day 10 of CNCC specification. *CDH2* and *GLI2* are necessary for mesenchymal identity and migration of CNCCs respectively. Regions annotated using hg19. (E) Heatmap of ZIC2 binding at regions that are not bound by ARID1A displaying a genomic relocation of ZIC2 in *ARID1A*-haploinsufficient conditions at day 10 of CNCC specification (*n* = 5,460). (F) Average profile of ZIC2 peaks at aberrantly accessible regions in CSS2 at day 10 of CNCC specification (*n* = 244). Center represents the average overlapping binding of ZIC2 at CSS-specific ATAC peaks across the genome. (G) Integrative Genomics Viewer example of gained ZIC2 binding in CSS2 relative to the isogenic CTRL2 at sites not bound by ARID1A at day 10 of CNCC specification. *RBFOX3* (NeuN) and *EOMES* (TBR2) are genes involved in neurogenesis. Regions annotated using hg19. (H) Pathways enriched from enhancers that are bound by ZIC2 in CSS2 at day 10 of CNCC specification and the respective −log_10_*p* value as determined by IPA. Pathways include those involved in neuronal signaling and processes.

To predict potential interactions between ARID1A and transcription factors in the regulation of EMT enhancers during CNCC specification, we performed a motif analysis on the ARID1A-bound enhancers and promoters. Regions that gained ARID1A binding and promoters that retained ARID1A binding both harbored conventional promoter motifs (Figures S4A and S4B), while the EMT enhancers that lose ARID1A binding in CSS cells revealed motifs for CNCC lineage-specific transcription factors^4^^,^^48^^,^^69^^,^^70^^,^^71^^,^^72^^,^^73^^,^^74^^,^^75^ (Figure 5A). Strikingly, nearly 40% of these enhancers harbored a ZIC family motif. *ZIC1* is not expressed at this developmental stage,^76^^,^^77^ while *ZIC2* and *ZIC3* are both highly expressed in day 10 CNCCs^56^^,^^78^^,^^79^^,^^80^^,^^81^ (Table S7). Reportedly, *ZIC2* variants cause holoprosencephaly type 5 in humans,^82^ a developmental syndrome that shares many phenotypic features^83^ with CSS.^30^^,^^31^^,^^32^ Therefore, we investigated ZIC2 as a potential interactor of ARID1A in the regulation of EMT.

To determine whether ZIC2 requires ARID1A to bind to EMT enhancers, we performed a ChIP-seq for ZIC2 in our isogenic CTRL and CSS lines. In *ARID1A*-haploinsufficient conditions, we observed attenuation of ZIC2 binding at EMT enhancers normally regulated by ARID1A (Figures 5B, 5C and S4C) that also exhibited loss of accessibility (Figure S4D). Similar to ARID1A, ZIC2 also preferentially bound enhancers in control conditions, and upon the loss of *ARID1A*, the global enhancer occupancy of ZIC2 was attenuated (Figures S4E and S4F). Specifically, enhancers for essential mesenchymal and CNCC factors, *CDH2* (N-cadherin) and *GLI2* displayed decreased ARID1A and ZIC2 binding in the CSS line of the isogenic pair (Figure 5D).

We further examined ZIC2 genome-wide occupancy and identified ∼5,500 ZIC2 peaks exclusive to the CSS line. These sites did not exhibit ARID1A binding even in CTRL conditions, indicating that *ARID1A*-haploinsufficiency triggered genomic relocation of ZIC2 to non-ARID1A regulated sites (Figures 5E and S4G). These sites of relocated ZIC2 binding were enriched for promoter motifs (Figure S4H) and displayed aberrant accessibility likely regulated by another BAF configuration or chromatin remodeler (Figures 5F and S4I).

Notably, ZIC2 was bound at regulatory regions for ∼21% of all the differentially upregulated genes in the CSS cell line. Genes associated with aberrantly bound ZIC2 enhancers (Figure 5G) were enriched for neuronal pathways (Figure 5H), while ZIC2-bound promoters were associated with common cellular processes (Figure S4J). Notably, ZIC2 protein levels were higher in CSS cell lines at day 10 of CNCC specification compared to CTRL (Figure S4K). These findings were consistent with the upregulation of neuronal genes observed in our *ARID1A*-haploinsufficient CSS lines. Collectively, these data suggest that ARID1A-BAF opened the chromatin at EMT enhancers during CNCC specification, allowing for ZIC2 binding. Furthermore, in the partial absence of *ARID1A*, ZIC2 relocated to neuronal enhancers whose accessibility was likely modulated by other chromatin remodelers.

### *Zic2* is required for NCC EMT and delamination *in vivo*

**Figure 6:**
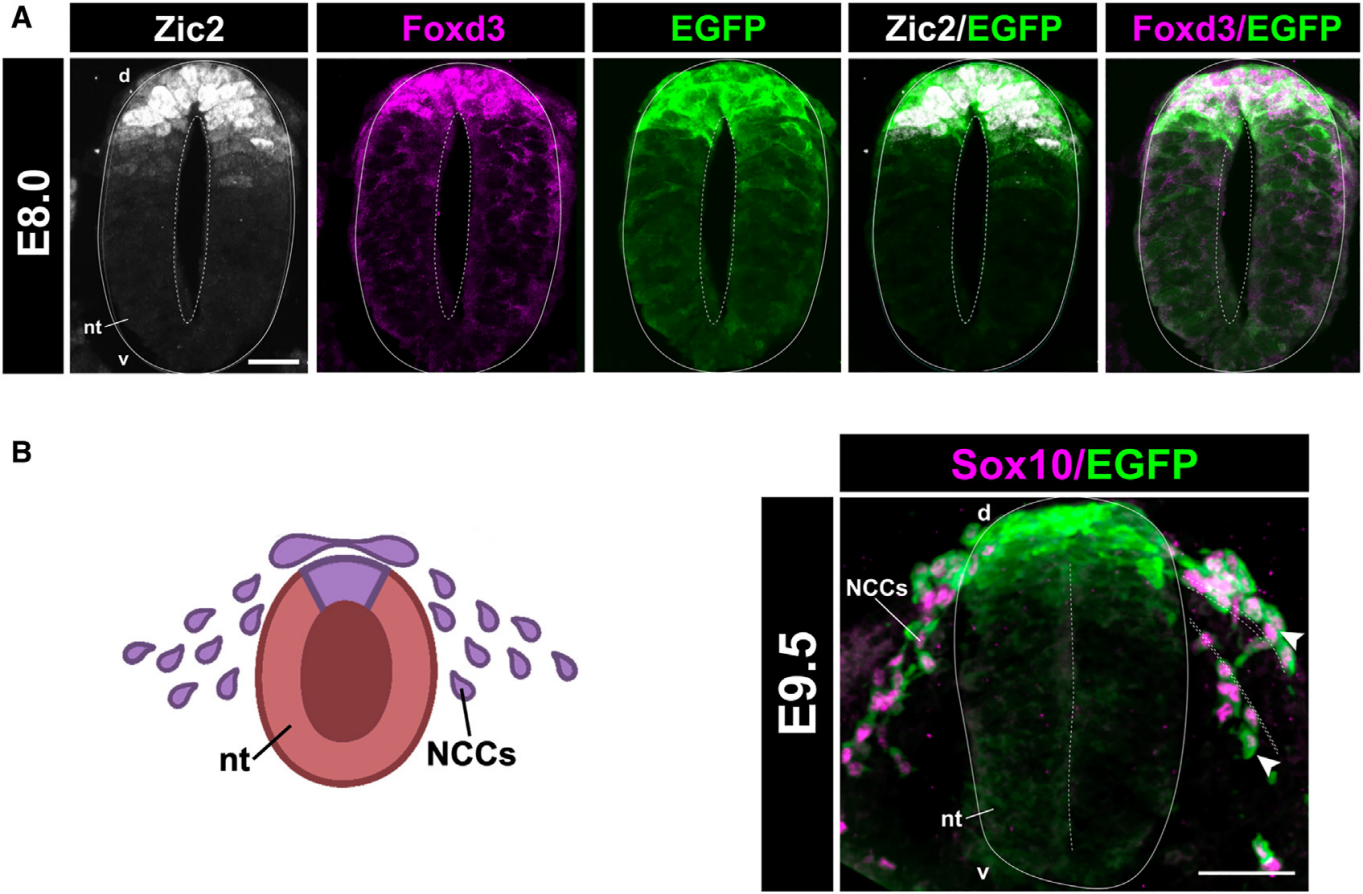
Zic2 activates neural crest EMT and delamination from the neural tube *in vivo* (A) A transverse cross-section of the neural tube in an E8.0 Tg(*Zic2*^EGFP^) mouse embryo immunostained for Zic2 and Foxd3. Scale bar, 20 μm, 40× magnification. (B) A schematic (left) and a transverse cross-section (right) of the neural tube in an E9.5 Tg(*Zic2*^EGFP^) mouse embryo immunostained for Sox10. White arrowheads highlight Sox10^+^ migrating NCCs are also GFP^+^ depicting migratory NCCs once expressed *Zic2* in a pre-migratory setting. Dotted lines show NCCs migrating in the dorsolateral/ventromedial paths. Scale bar, 50 μm, 40× magnification. d, dorsal; v, ventral; nt, neural tube; NCCs, neural crest cells. Schematic made with BioRender.com.

To further investigate the role of Zic2 in EMT and NCC formation *in vivo*, we characterized the spatiotemporal levels of Zic2 during murine neurulation using a previously described *Zic2* reporter mouse line, Tg(*Zic2*^EGFP^).^54^ At E8.0, prior to CNCC delamination and migration (similar to the pre-migratory stage *in vitro*), Zic2 signal is detectable all along the dorsal neural tube in cells positive for Foxd3, a marker of pre-migratory NCCs (Figure 6A). By E9.5, there were few Zic2^+^ pre-migratory cells remaining in the dorsal region of the neural tube, indicating successful NCC delamination and migration (Figure 6B; right). Additionally, we observed migratory NCCs (Sox10^+^/EGFP) migrating throughout the dorsolateral and ventromedial paths in the mesenchyme (Figure 6B; right). This observation suggested that Sox10/EGFP^+^ cells migrating in the mesenchyme (Figure 6B; right) were previously Zic2^+^ pre-migratory cells originating from the dorsal region of the neural tube (Figure 6A).

**Figure 7:**
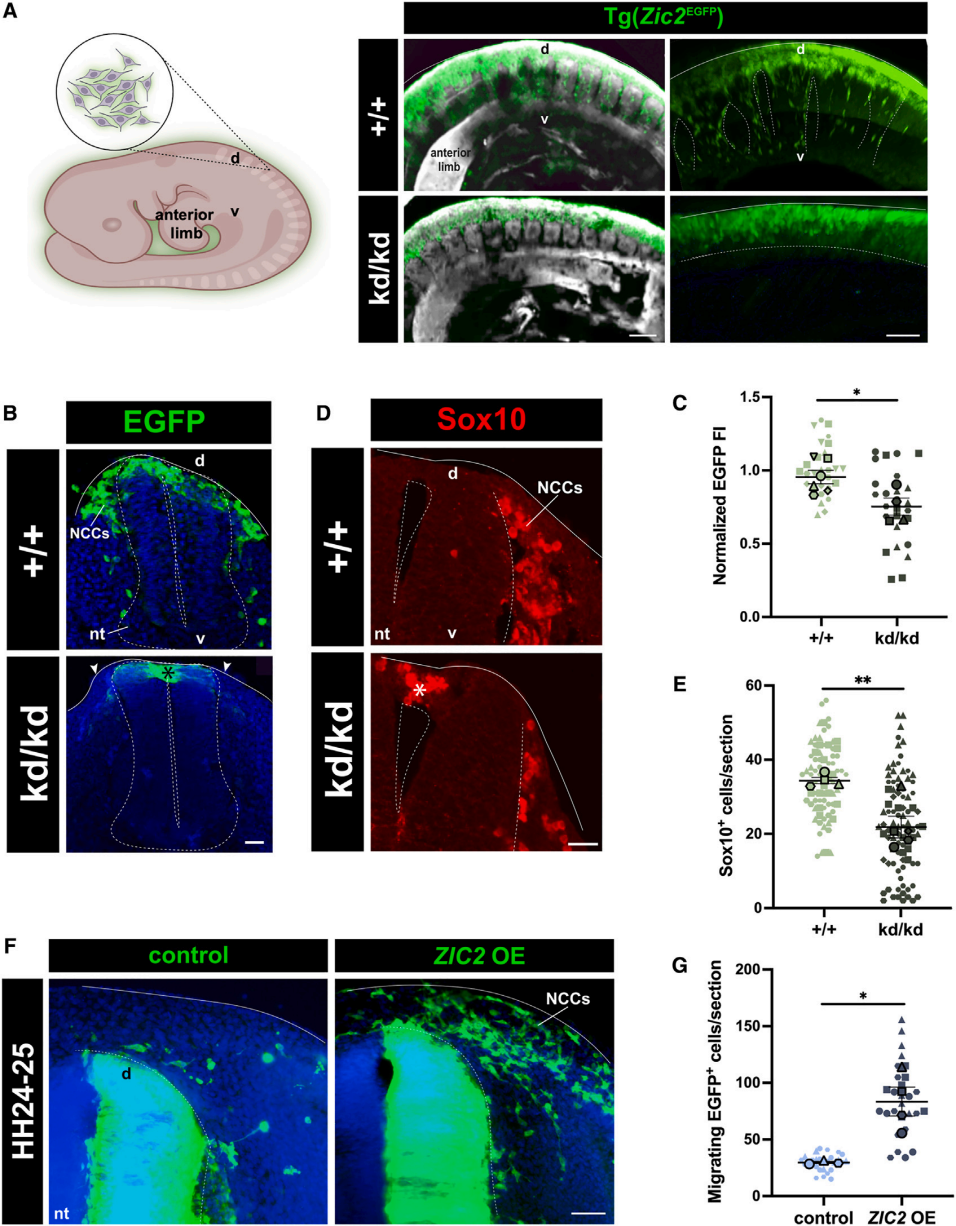
*Zic2* perturbation prevents NCC migration, while *ZIC2* overexpression induces ectopic NCC delamination from the neural tube *in vivo* (A) Schematic (left) and iDISCO (right) of the lateral view of E9.5 whole-mount control [*Zic2*^+/+^;Tg(*Zic2*^EGFP^)] and *Zic2*-mutant [*Zic2*^kd/kd^;Tg(*Zic2*^EGFP^)] mouse embryos displaying lack of migration into the mesenchyme in *Zic2*-mutant conditions. Scale bar, 100 μm, 5× magnification. Schematic made with BioRender.com. (B) Representative images of a transverse cross-sections of the neural tube from E9.0 control [*Zic2*^+/+^; Tg(*Zic2*^EGFP^)] and *Zic2*-mutant [*Zic2*^kd/kd^;Tg(*Zic2*^EGFP^)] mouse embryos. Arrowheads highlight the absence of GFP^+^ cells in the dorsal sub-epidermal area of a *Zic2*-mutant embryo, with GFP^+^ cells accumulating inside the dorsal neural tube represented by an asterisk. Scale bar, 10 μm, 40× magnification. (C) Superplot quantification of the fluorescent intensity (FI) of GFP^+^ cells in the dorsal sub-epidermis of E9.0 [*Zic2*^+/+^; Tg(*Zic2*^EGFP^)] and [*Zic2*^kd/kd^; Tg(*Zic2*^EGFP^)] mouse embryos normalized to the control intensity. *n* = 4–6 independent experiments represented by a distinctive shape with each small data point representing at least four sections per embryo and 4–6 embryos per genotype. The larger data points correspond to the average values of each replicate. A two-tailed unpaired t test performed on the average values between *Zic2*-WT and *Zic2*-mutant embryos; *p* < 0.05 was considered significant; ^∗^*p* = 0.0255. Error bars represent the standard error of the mean. (D) Representative images of a transverse cross-sections of the neural tube from E10.5 control [*Zic2*^+/+^;Tg(*Zic2*^EGFP^)] and *Zic2*-mutant [*Zic2*^kd/kd^;Tg(*Zic2*^EGFP^)] mouse embryos immunostained for Sox10. The asterisk highlights an aberrant accumulation of Sox10^+^ cells inside the neural tube at this developmental stage. Scale bar, 20 μm, 40× magnification. (E) Superplot quantification of the number of Sox10^+^ cells per section. *n* = 4–5 independent experiments represented by a distinctive shape with each small data point representing at least five sections per embryo and 4–5 embryos per genotype. The larger data points correspond to the average values of each replicate. A two-tailed unpaired t test performed on the average values between *Zic2*-WT and *Zic2*-mutant mouse embryos; *p* < 0.05 was considered significant; ^∗∗^*p* < 0.0074. Error bars represent the standard error of the mean. (F) Transverse cross-sections of the developing neural tube at HH24-25 in chick embryos electroporated at HH9-11 with plasmids carrying *ZIC2*-GFP or GFP alone. OE, overexpression. Scale bar, 50 μm, 10× magnification. (G) Superplot quantification of the number of migrating GFP^+^ cells in sections of HH24-25 chick embryos electroporated at HH9-11 with plasmids carrying *ZIC2*-GFP or GFP alone. *n* = 3–4 independent experiments represented by a distinctive shape with each small data point representing at least five sections per embryo and 3–4 embryos per genotype. The larger data points correspond to the average values of each replicate. A two-tailed unpaired t test performed on the average values between control-GFP and *ZIC2*-GFP; *p* < 0.05 was considered significant; ^∗^*p* < 0.0161. Error bars represent the standard error of the mean. D, dorsal; v, ventral; nt, neural tube; NCCs, neural crest cells.

In order to assess the impact of *Zic2* loss-of-function in NCCs, we crossbred the Tg(*Zic2*^EGFP^) strain with the previously reported *Zic2*^kd^ line^56^ to obtain mutant [*Zic2*^kd/kd^;Tg(*Zic2*^EGFP^)] and control [*Zic2*^+/+^;Tg(*Zic2*^EGFP^)] embryos in the same litter. In *Zic2*-WT embryos, many EGFP-labeled cells exhibited a stereotypical migration pattern through the dorsal sub-epidermal region of the mesenchyme (Figures 7A–7C). However, in the *Zic2*-mutant embryos, very few migrating EGFP-labeled cells were detected (Figures 7A–7C). Instead, an aberrant accumulation of EGFP^+^ cells was observed in the dorsal neural tube in the mutants compared to the controls (Figure 7B). Notably, there was a significant reduction of migratory Sox10^+^ cells detected in the *Zic2*-mutant embryos (Figures 7D and 7E). The Sox10^+^ cells were mostly restricted to the dorsal neural tube in the *Zic2*-mutant embryos indicating a lack of EMT, delamination, and migration in the absence of *Zic2* (Figure 7D). Consistent with our *in vitro* data, these results indicate that NCCs failed to delaminate from the neural tube in *Zic2*-mutant embryos, demonstrating a crucial role for Zic2 in cell delamination via EMT.

### *ZIC2* gain-of-function at pre-migratory stages elicits aberrant NCC delamination *in vivo*

To explore whether Zic2 was sufficient to trigger cell detachment through EMT *in vivo*, we conducted gain-of-function experiments by ectopically expressing human *ZIC2* and/or GFP into the neural tube of HH9-11 chick embryos (pre-neurulation). As expected, GFP^+^ cells were largely observed in the neural tube at developmental stage HH24-25 (Figure 7F). Notably, ectopic expression of *ZIC2* at the same developmental stages triggered a significant increase in the number of cells delaminating from the neural tube (Figures 7F and 7G). This indicates that ectopic expression of *ZIC2* was sufficient to induce delamination in NCCs. Collectively our *in vivo* gain-of-function data further support a key role for ZIC2 in EMT and delamination of NCCs from the neural tube.

## Discussion

ARID1A is one of two integral DNA-binding subunits of the canonical BAF chromatin remodeling complex. While ARID1A-BAF has a wide-range of roles including regulating aberrant chromatin accessibility in cancer contexts,^6^^,^^84^ its function in physiological craniofacial development is underappreciated. Variants in *ARID1A* are associated with CSS, a rare developmental disorder predominantly characterized by coarse craniofacial features.^31^^,^^32^ The hyperplastic craniofacial skeletal features seen in CSS are derived from the cranial neural crest.^31^^,^^33^^,^^37^

Herein, we investigated the role of ARID1A-BAF in the molecular regulation of CNCC formation using *ARID1A*^+/−^ patient-derived iPSCs. It is critical to highlight the considerable incidence of somatic mosaicism associated with *ARID1A* variants.^17^^,^^18^ While *de novo* germline mutations have been reported, most individuals with CSS who harbor variants in *ARID1A* are typically mosaic.^60^ Recently, it was shown that germline mutations in *ARID1A* result in a much more severe phenotype, often with multiple congenital anomalies, frequently leading to termination of pregnancy or spontaneous demise during or after birth.^60^ In a research context, this mosaicism provides us with an isogenic cellular platform in which the wild-type and *ARID1A*^+/−^ lines share the same genetic background. This offers the advantage of eliminating confounding genomic factors, ensuring all the cellular phenotypes observed are a direct result of *ARID1A* haploinsufficiency from a clinical setting.

We previously reported an essential ARID1 subunit switch between ARID1A and ARID1B in BAF that occurs at the transition of iPSC to neuroectoderm and neuroectoderm to CNCC.^3^ Notably, subunit switching for distinct regulatory function is not restricted to BAF as this phenomenon occurs in other chromatin remodelers. For example, among the mutually exclusive ATPases in the NuRD complex, CHD3/4/5, each subunit coordinates specific sets of genes necessary for various stages of cortical development.^85^ Ultimately, signaling pathways and interactors that regulate these ARID1 subunit switches, and its timing remain unknown. Similarly, recruitment of BAF to its target regions is globally regulated by the AP-1 transcription factor complex.^71^ However, this recruitment process could also be guided by cell type-specific transcription factors essential for NCC formation^48^^,^^72^^,^^73^^,^^74^ as depicted in our motif analysis of ARID1A-bound regions (Figure 5A).

In this study, we have unveiled an essential axis for CNCC formation, including ZIC2 and ARID1A-BAF. ZIC2 is a transcription factor of the GLI superfamily, which is known to regulate several aspects of neurodevelopment.^86^^,^^87^^,^^88^ Variants of *ZIC2* are associated with holoprosencephaly type 5,^82^ a developmental syndrome that shares many craniofacial phenotypes^83^ with CSS.^32^ Individuals with holoprosencephaly harboring *ZIC2* variants present with bitemporal narrowing, upslanting palpebral fissures, a short nose with anteverted nares, a broad and well demarcated philtrum, and large ears.^83^ Notably, these specific craniofacial anomalies do not appear in individuals with pathogenic variants in other holoprosencephaly-associated genes.^83^

Our *in vitro* and *in vivo* data, encompassing human iPSCs, mouse models, and chick models, support a mechanism in which ARID1A opens the chromatin for ZIC2 at the onset of EMT to induce NCC delamination from the neural tube. With multiple *in vivo* models, we demonstrate that *Zic2* is indispensable for NCC EMT and subsequent migration, and validate the molecular foundation of this regulation through ARID1A-mediated accessibility at EMT enhancers *in vitro*. Supporting this model *in vivo*, Zic2 is present in mouse pre-migratory NCCs and is required for their delamination from the neural tube. Conversely, ectopic expression of *ZIC2* in chick embryos displays asynchronous delamination and EMT.

In *ARID1A*^+/−^ conditions, accessibility at EMT enhancers is perturbed, preventing lineage-specific transcription factors, like ZIC2, from binding at these elements to promote EMT. In the partial absence of *ARID1A*, ZIC2 relocates to neuronal enhancers, suggesting ZIC2 plays a role in the aberrant activation of neuronal networks. As both NCCs and neurons are derived from the neuroectoderm,^35^^,^^89^^,^^90^^,^^91^^,^^92^ and neuroectoderm-like cells are successfully specified in *ARID1A*-haploinsufficient conditions, our results potentially suggest a default trajectory toward the neuronal lineage when the neural crest lineage cannot be specified. However, further studies will be needed to validate this model.

ZIC2 has multifaceted functions in neuronal development,^86^^,^^87^^,^^88^ hindbrain patterning,^78^^,^^93^ and axon guidance.^54^^,^^94^^,^^95^^,^^96^ Zic2 controls axon midline repulsion, and this neurodevelopmental process has been recently proposed to be influenced by enhancers containing Zic2 binding motifs.^97^ Thus, the genomic relocation of ZIC2 and aberrant upregulation of neuronal-associated genes is consistent with its known role in regulating neuronal processes. However, additional studies will be required to determine the molecular regulation and contribution from other lineage-specific transcription factors and chromatin remodelers underlying this relocation.

Notably, EMT is a main function of other non-developmental processes including wound healing^98^ and fibrosis.^99^^,^^100^ Therefore, it would be crucial to investigate whether ARID1A-BAF directs EMT in these settings and if distinctive cell-type-specific transcription factors are involved. Additionally, EMT extensively modulates cancer progression and metastasis.^101^ *ARID1A* is mutated in ∼10% of all tumors with postulated balancing oncogenic^20^^,^^21^ and tumor suppressive functions.^22^^,^^23^^,^^24^^,^^25^^,^^84^ However, ARID1A is predominantly reported to inhibit EMT in a cancer context.^102^^,^^103^^,^^104^ This may suggest a physiological role for ARID1A-BAF in promoting EMT in embryonic development, but further studies will be needed to support this hypothesis.

In summary, this study provides crucial insight into how chromatin remodelers regulate neural crest specification, delamination, and migration, suggesting a potential pathological mechanism underlying severe craniofacial phenotypes in certain developmental syndromes.

## Data and code availability

The accession number for the RNA-seq, ChIP-seq, and ATAC-seq data reported in this paper is GEO: GSE261846.

## Acknowledgments

The authors are grateful to the families of the individuals with CSS for donating the samples and to Sandra Deliard and Alessandro Gardini for the use of the IPA software and for insightful comments. The authors thank the Genomic Facility at The Wistar Institute (Philadelphia, PA) for the Next Generation Illumina Sequencing. For this work, M.T. was funded by the 10.13039/100011671G. Harold and Leila Y. Mathers Foundation. E.H. was funded by 10.13039/501100004837MCIN (PID2022-138245NB-I00), the 10.13039/100010434'la Caixa' Foundation (HR21-00824), 10.13039/501100003359Generalitat Valenciana (PROMETEO/2020/007), and the Severo Ochoa Program for Centers of Excellence (CEX2021-001165-S). S.B.M. was funded by the 10.13039/100000002National Institutes of Health (NIH R01 R01ES033197).

## Author contributions

M.T., E.H., and S.M.B. designed the project. S.M.B. performed all the human iPSC and CNCC experiments and analyzed all the genomic data. A.G.d.G., C.M.-P., and M.T.L.-C performed all the *in vivo* experiments. C.S. performed all the iPSC and CNCC immunofluorescence and contributed to data analysis. F.J.W., M.E.C., and J.K. contributed to some of the experiments. G.W.E.S. recruited the individuals with CSS and obtained the skin fibroblasts. H.M.M.M. reprogrammed the CSS patient-derived fibroblasts into iPSCs and performed quality checks. K.A.P., D.T., S.B.M., and S.A.B. contributed to data analysis and offered critical support. S.M.B. and M.T. analyzed the data and wrote the manuscript. All the authors read and approved the manuscript.

## Declaration of interests

The authors declare no competing interests.

## Supplemental information

Document S1. Figures S1–S4

Table S1. Spreadsheet of all antibodies used

Table S2. Differentially expressed genes at the iPSC stage in CSS lines relative to CTRL lines

Table S3. TPMs of annotated genes in iPSCs for CTRL and CSS lines

Table S4. TPMs of annotated genes CTRL and CSS lines cultured in CNCC late maintenance media

Table S5. Differentially expressed genes at the CNCC stage in CSS lines relative to CTRL lines (cultured in early maintenance media)

Table S6. Differentially expressed genes in CSS lines relative to CTRL lines in D10 CNCCs

Table S7. TPMs of annotated genes for CTRL and CSS lines at day 10 of CNCC specification

Document S2. Article plus supplemental information
