# Supplementary material for: ARID1A-BAF coordinates ZIC2 genomic occupancy for epithelial-to-mesenchymal transition in cranial neural crest specification
Source: Am J Hum Genet. 2024 Sep 2;111(10):2232–52. doi: 10.1016/j.ajhg.2024.07.022 (PMC11480806; doi:10.1016/j.ajhg.2024.07.022)
Supplement: Document S1. Figures S1–S4 [file mmc1.pdf]

The American Journal of Human Genetics, Volume *111*

Supplemental information

ARID1A-BAF coordinates ZIC2 genomic occupancy for epithelial-to-mesenchymal transition in cranial neural crest specification

Samantha M. Barnada, Aida Giner de Gracia, Cruz Morenilla-Palao, Maria Teresa López-Cascales, Chiara Scopa, Francis J. Waltrich Jr., Harald M.M. Mikkers, Maria Elena Cicardi, Jonathan Karlin, Davide Trotti, Kevin A. Peterson, Samantha A. Brugmann, Gijs W.E. Santen, Steven B. McMahon, Eloísa Herrera, and Marco Trizzino

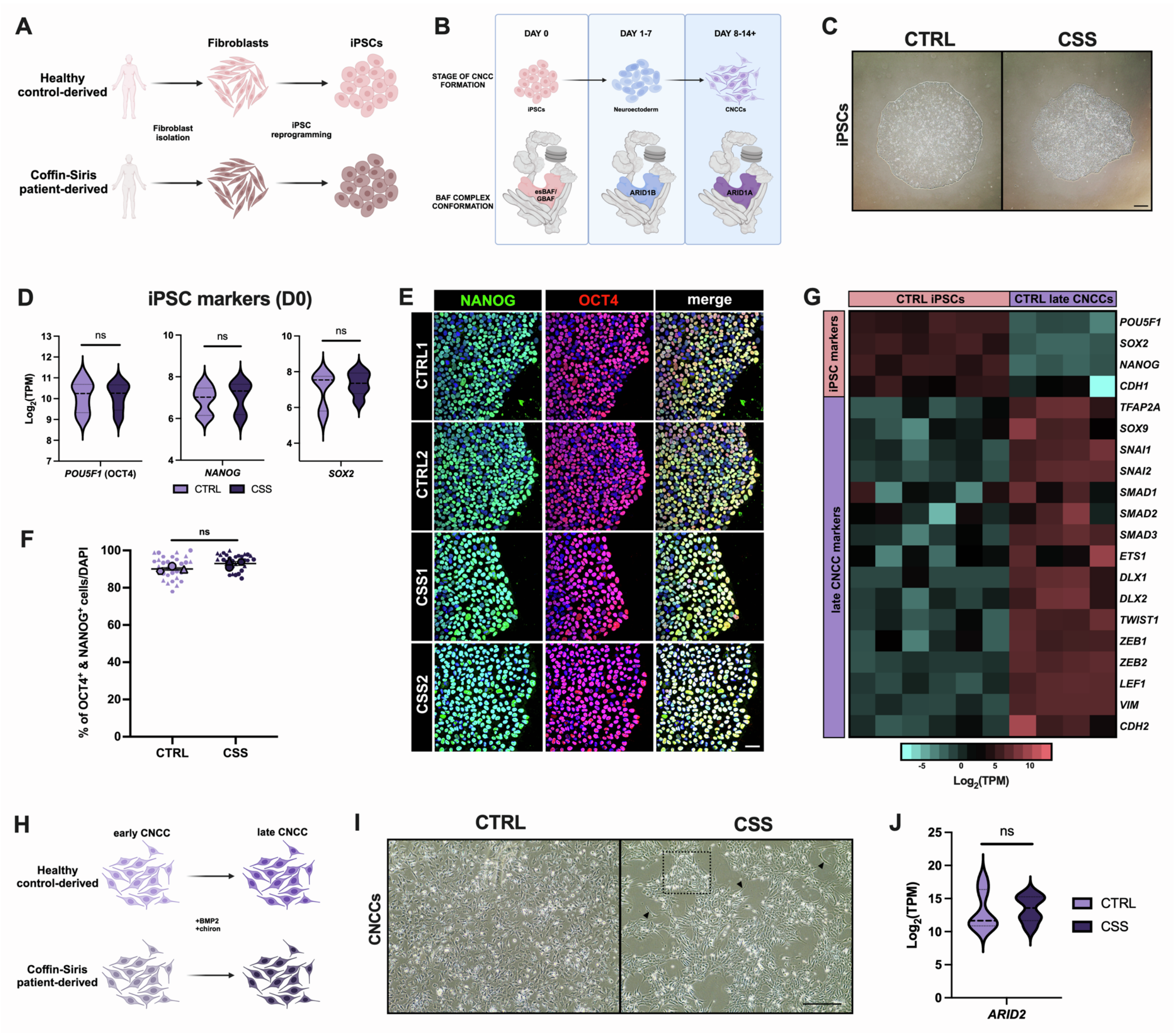

Figure S1 – Validation of iPSC reprogramming and *in vitro* CNCC specification in CTRL and CSS cell lines. (A) Graphical illustration of iPSC reprogramming from *ARID1A*-haploinsufficient Coffin-Siris Syndrome patient-derived tissue and healthy control tissue. Made with BioRender.com. (B) Visual schematic of ARID1 subunit switching at different morphological stages throughout CNCC specification. Made with BioRender.com. (C) Representative brightfield images of CTRL and CSS iPSC colonies show no difference in cellular morphology. Scale bar = 300µm, 4X magnification. (D) Violin plots displaying $\log_2$(TPM) of iPSC markers, *POU5F1* (OCT4), *NANOG*, and *SOX2* in CTRL and CSS iPSCs. There is no significant difference in expression of pluripotent factors between CTRL and CSS iPSCs. A two-tailed unpaired t-test was performed and $p < 0.05$ was considered significant; *POU5F1* (OCT4): ns $p = 0.992$, *NANOG*: ns $p = 0.7227$, and *SOX2*: ns $p = 0.5268$. (E) Representative images and (F) quantification of an immunofluorescence for pluripotency factors OCT4 and NANOG performed in CTRL and CSS iPSCs. (E) DAPI staining on nuclei in blue. Images shown are taken at 20X magnification; scale bar = 50µm. (F) SuperPlot quantification of percentage of OCT4/NANOG double-positive cells per DAPI. n = 3 represented by a distinctive shape with each small data point representing a captured image. The larger data points correspond to the average values of each replicate. A two-tailed unpaired t-test performed on the average values between CTRL and CSS lines; $p < 0.05$ was considered significant; ns $p = 0.0894$. (G) Heatmap of the expression of pluripotent markers and

CNCC markers in CTRL iPSCs (D0) and late specified CNCCs (+BMP2 and CHIR-99021). Pluripotent genes, including *POU5F1* (OCT4), are deactivated while CNCC specifiers are upregulated in CNCCs relative to iPSCs. iPSC columns represent 3 individual replicates for CTRL1 and CTRL2 and the late CNCC columns represent 2 individual replicates from 2 separate CNCC specifications for CTRL1 and CTRL2. (H) Schematic of "early" to "late" CNCC specification through the addition of exogenous BMP2 and CHIR-99021 (GSK3 inhibitor). Made with BioRender.com. (I) Representative brightfield images of D14 CNCCs demonstrating altered morphology of CSS CNCCs compared to CTRLs. Black arrows point to examples of aberrant elongated morphology. Black dotted box highlights an example of abnormal cell clustering. Scale bar = 300μm, 10X magnification. (J) Violin plots displaying $\log_2$(TPM) expression of *ARID2* at D10 of CNCC specification. There is no significant difference in expression between CTRL and CSS lines. A two-tailed unpaired t-test was performed and $p < 0.05$ was considered significant; $p = 0.8420$. Error bars represent standard error of the mean.

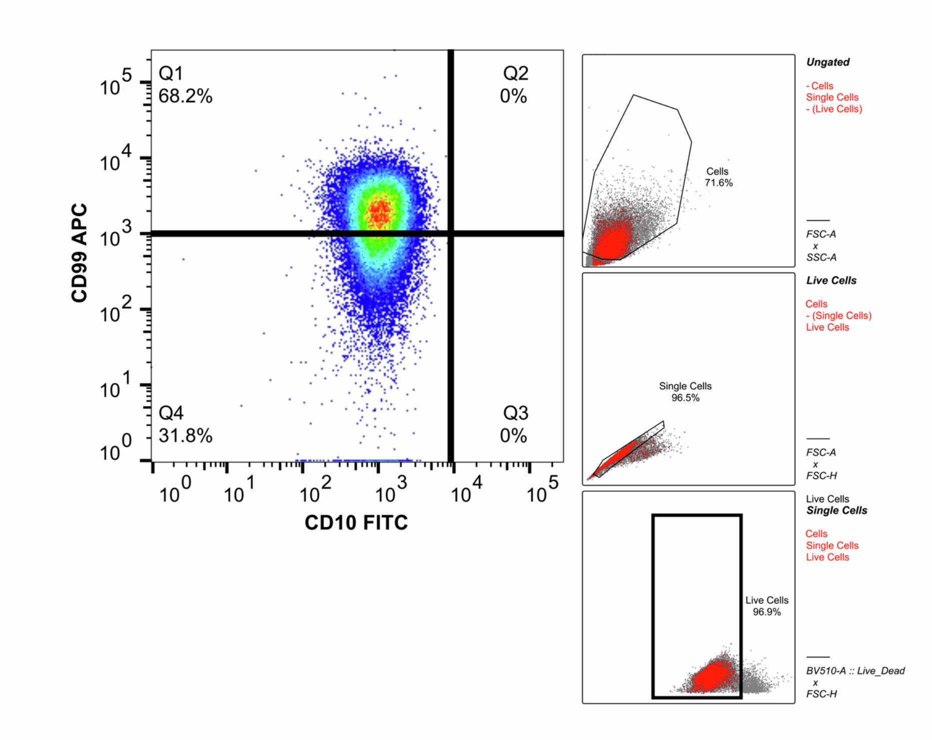

Figure S2 – Flow cytometry back-gating analysis for Figure 1C. Forward scatter area (FSC-A) versus side scatter area (SCC-A) was used to select the population of cells. FSC-A versus forward scatter height (FSC-H) was used to select single cells and exclude doublets. Subsequent gating was used to identify stained live cells (BV510-A vs FSC-H). CNCC-positive populations were characterized by CD99-APC versus CD10-FITC surface marker expression.

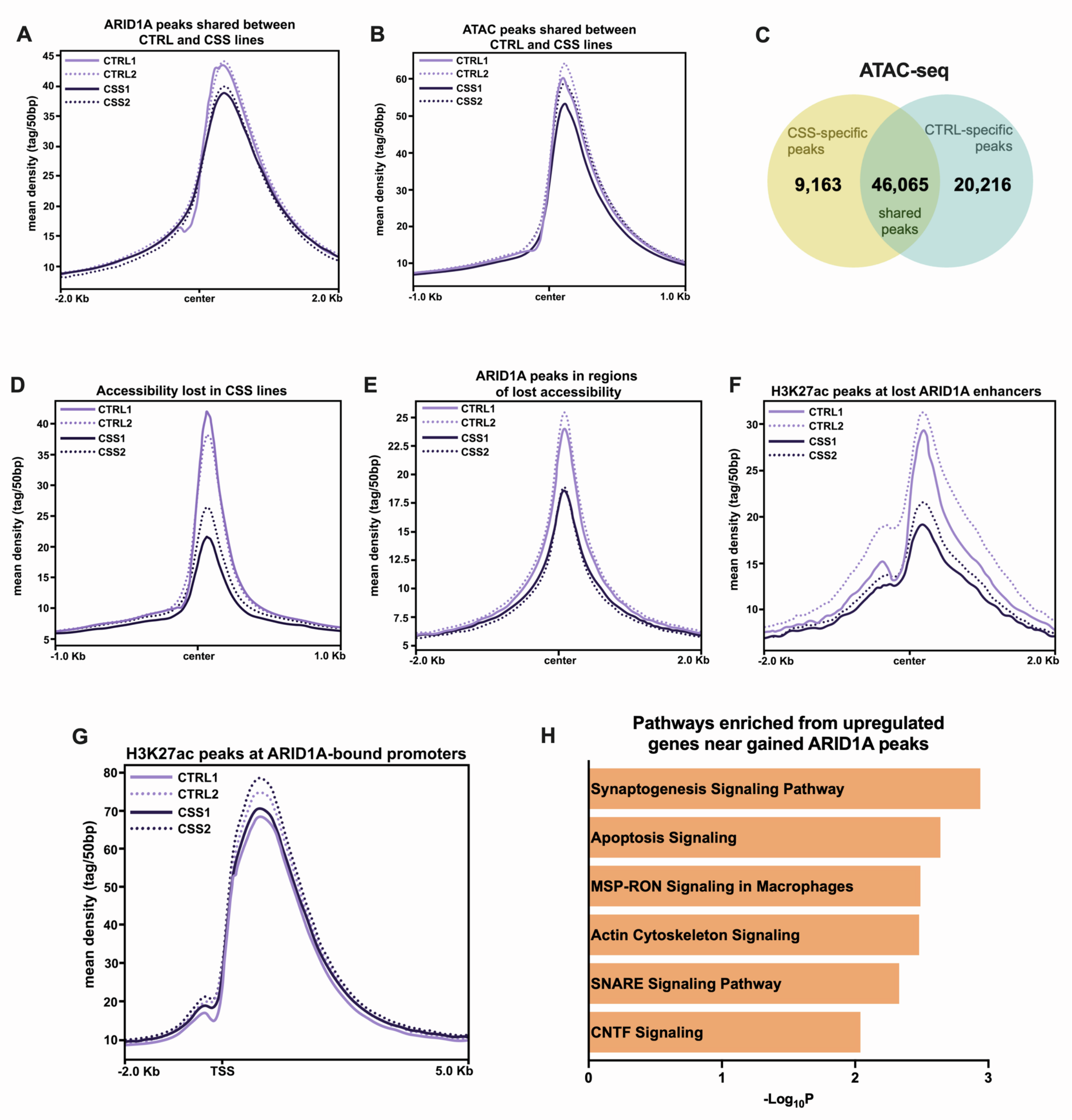

Figure S3 – Global ARID1A binding and accessibility across CTRL and CSS D10 CNCCs. (A) Average profile of ARID1A peaks shared between CTRL and CSS lines at D10 of CNCC specification (n = 21,724). Center represents the average overlapping shared binding of ARID1A across the genome. (B) Average profile of regions of accessibility (ATAC-seq peaks) conserved between CTRL and CSS lines at D10 of CNCC specification (n = 46,065). Center represents the average overlapping shared ATAC-seq peaks across the genome. (C) Venn diagram displaying the number of CSS-specific ATAC-seq peaks (ATAC peaks “gained” in CSS cell lines; 9,163), CTRL-specific ATAC-seq peaks (ATAC peaks “lost” in CSS lines; 20,216), and shared ATAC-seq peaks between CTRL and CSS cell lines (conserved regions of accessibility; 46,065) via an ATAC-seq performed at D10 of CNCC specification. (D) Average profile of ATAC peaks lost in CSS lines at D10 of CNCC specification (n = 20,216). Center represents the average overlapping lost ATAC peaks across the

genome. (E) Average profile of ARID1A peaks at regions of lost accessibility in the CSS lines at D10 of CNCC specification (n = 20,216). Center represents the average overlapping binding of ARID1A at regions of lost accessibility across the genome. (F) Average profile of H3K27ac ChIP-seq signal at enhancer regions that lose ARID1A binding and H3K27ac signal in the CSS cell lines at D10 of CNCC specification (n = 1,925). Center represents the average overlapping lost CSS H3K27ac and ARID1A peaks at enhancer regions across the genome. (G) Average profile of H3K27ac peaks at promoter regions that retain ARID1A binding and H3K27ac ChIP-seq signal in the CSS cell lines at D10 of CNCC specification (n =7,724). TSS represents the average overlapping H3K27ac ChIP-seq signal at ARID1A-bound transcription start sites throughout the genome. (H) Pathways enriched from differentially upregulated genes that are nearest to a gained ARID1A peak in the CSS lines at D10 of CNCC specification and the respective $-\log_{10}$p-value as determined by IPA.

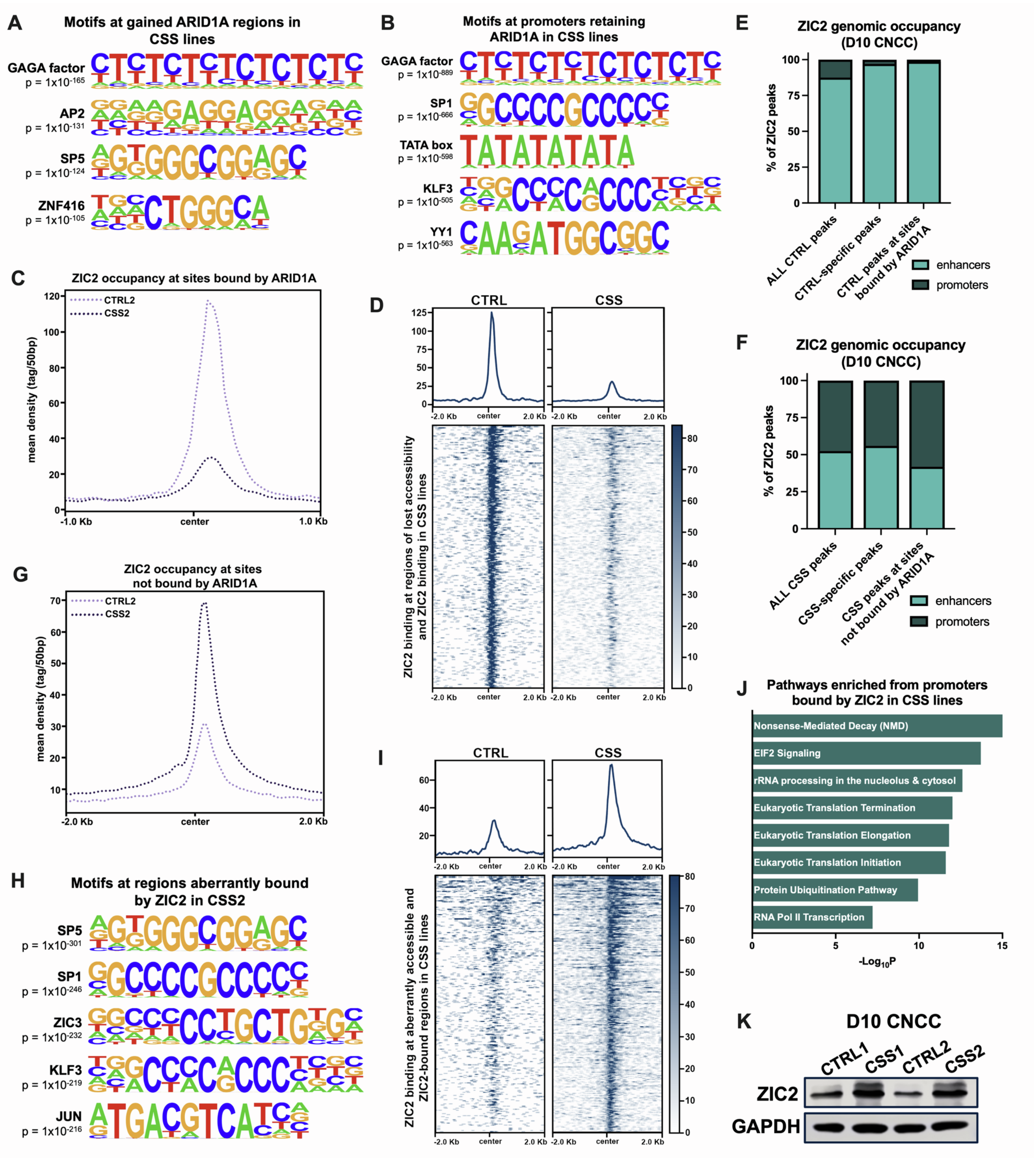

Figure S4 – Genomic features of ARID1A-bound promoters and ZIC2 occupancy and expression at D10 of CNCC specification. (A) Motif analysis at gained ARID1A peaks in the CSS lines identified by HOMER. (B) Motif analysis at retained ARID1A-bound promoters in CSS lines identified by HOMER. (C) Average profile of ZIC2 ChIP-seq peaks at ARID1A sites at D10 of CNCC specification in the isogenic system (n = 358). Center represents the average overlapping binding of ZIC2 at ARID1A-bound regions across the genome. (D) Heatmap of ZIC2 binding at regions that lose accessibility and ZIC2 binding in the CSS lines at D10 of CNCC specification (CTRL-specific; n = 232). (E) Stacked bar plot depicting the percentage of ZIC2 ChIP-seq peaks enriched at enhancers (>1kb from the closest transcription start site or TSS) and promoters (<1kb from the

closest TSS) at all CTRL ZIC2 ChIP-seq peaks (including peaks shared with CSS lines; 87% at enhancers, 13% at promoters), CTRL-specific ChIP-seq peaks (ZIC2 peaks exclusive to the CTRLs; 97% at enhancers, 3% at promoters), and overlapping ZIC2- and ARID1A-bound sites in CTRLs (99% at enhancers, 1% at promoters). (F) Stacked bar plot depicting the percentage of ZIC2 peaks enriched at enhancers (>1kb from the closest transcription start site or TSS) and promoters (<1kb from the closest TSS) at all CSS ZIC2 ChIP-seq peaks (including peaks shared with the CTRL line; 52% at enhancers, 48% at promoters), CSS-specific peaks (ZIC2 ChIP-seq peaks exclusive to the CSS cell line; 56% at enhancers, 44% at promoters), and ZIC2 binding in the CSS line at regions not bound by ARID1A (42% at enhancers, 58% at promoters). (G) Average profile of ZIC2 ChIP-seq peaks at sites not bound by ARID1A at D10 of CNCC specification in the isogenic system (n = 5,460). Center represents the average overlapping binding of ZIC2 at non-ARID1A bound regions across the genome. (H) Motif analysis via HOMER of regions bound by ZIC2 in CSS2 but not normally bound by ARID1A in CTRLs. (I) Heatmap of ZIC2 binding at regions that are aberrantly accessible and display a genomic relocation of ZIC2 in *ARID1A*-haploinsufficient conditions at D10 of CNCC specification (n = 244). (J) Pathways enriched from promoters that are bound by ZIC2 in CSS2 at D10 of CNCC specification and the respective $-\log_{10}$p-value as determined by IPA. Pathways include those necessary for basic cellular functions and survival including transcription, translation, and nonsense-mediated decay. (K) Immunoblot of ZIC2 at D10 of CNCC specification between CTRL and CSS cell lines. An increase of ZIC2 levels in the CSS lines is consistent with genomic relocation of ZIC2 to neuronal enhancers.
